# Comparative Safety of PD-1/PD-L1 Inhibitors for Cancer Patients: Systematic Review and Network Meta-Analysis

**DOI:** 10.3389/fonc.2019.00972

**Published:** 2019-10-01

**Authors:** Ya-fang Huang, Wen-jie Xie, Hai-yu Fan, Juan Du

**Affiliations:** ^1^School of General Practice and Continuing Education, Capital Medical University, Beijing, China; ^2^Department Clinical Research, University of Bern, Bern, Switzerland; ^3^Center of Stroke, Beijing Institute for Brain Disorders, Capital Medical University, Beijing, China

**Keywords:** PD-1 inhibitors, PD-L1 inhibitors, treatment-related adverse events, immune-related adverse events, network meta-analysis

## Abstract

**Background:** Comprehensive evidence comparing treatment-related adverse events (trAEs) among PD-1/PD-L1 inhibitors is unavailable.

**Methods:** A systematic review and network meta-analysis (NMA) was conducted. Randomized controlled trials in cancer patients treated with PD1/PD-L1 inhibitors or their combinations with chemotherapy/placebo and compared with PD1/PD-L1 inhibitors/chemotherapy/placebo were identified through comprehensive searches of multiple databases. Bayesian NMA was performed using random-effects model. Relative ranking of treatments was assessed with surface under the cumulative ranking (SUCRA) probabilities. Incidences and odds ratios of trAEs and immune-related adverse events (irAEs) of all-grade (Grade 1–5) and high-grade (Grade 3–5) were estimated.

**Results:** Twenty-three RCTs (14,204 patients) comparing six different strategies were included. The incidence of trAEs was lowest for PD-L1 inhibitors (all-grade: pooled incidence = 60.4%, SUCRA = 77.2%; high-grade: 6.4, 73.8%). PD-L1 inhibitors plus chemotherapy had the highest incidence of all-grade trAEs (88.6, 10.1%), while PD-1 inhibitors plus chemotherapy had the highest incidence of high-grade trAEs (8.2, 9.3%). The use of PD-1/PD-L1 inhibitors alone was associated with significant reductions on high-grade trAEs, compared with PD-1/PD-L1 inhibitors plus chemotherapy. PD-1 inhibitors had the highest incidence of irAEs (all-grade: 15.1, 9.5%; high-grade: 3.5, 16.8%). Compared with PD-L1 inhibitors, PD-1 inhibitors neither increased trAEs nor irAEs significantly. Results from sensitivity analyses were consistent.

**Conclusions:** Current data showed that PD-L1 inhibitors had the best safety on both trAEs and irAEs. Awareness of the comparative safety could promote further appropriate utilization of PD-1/PD-L1 inhibitors in clinical practice.

## Introduction

Programmed cell death 1 (PD-1) and programmed cell death ligand 1 (PD-L1) inhibitors have shown clinical activity and marked efficacy in metastatic cancer therapy (1–6). Over recent years, many PD-1/PD-L1 inhibitors have been approved by Food and Drug Administration (FDA). For example, nivolumab and pembrolizumab (both PD-1 inhibitors) were approved for non-small cell lung cancer (NSCLC) and metastatic melanoma treatment (7–10). Atezolizumab and durvalumab were approved for NSCLC and urothelial carcinoma treatment (11–14). These regulatory approvals have resulted in a widespread prescribing of PD-1/PD-L1 inhibitors in real-world clinical practice. However, PD-1/PD-L1 inhibitors could disrupt normal immune tolerance mechanisms, thus lead to immune-related adverse events (irAEs) and other treatment-related adverse events (trAEs) (15–18).

The improvements in marked clinical efficacy should be balanced against the potentially serious or life-threatening adverse events when choosing among different therapeutic regimens. It is important for clinicians to be fully aware of the treatment-related risks and to better manage cancer treatment. To date, numerous randomized controlled trials (RCTs) have been conducted to investigate the efficacy and safety profiles of PD-1/PD-L1 inhibitors (2, 5, 6, 19–23). However, RCTs that are not prospectively designed with the particular safety outcome as a primary endpoint may not have sufficient sample size to detect important trAEs and irAEs. Thus, they would lack more convincing statistical power or could not reliably evaluate the potentially increased risks caused by PD-1/PD-L1 inhibitors. Moreover, head-to-head RCT, that compares PD-1/PD-L1 inhibitors against one another, is not available (24). Comparative trAEs and irAEs among different PD1/PD-L1 inhibitor-related therapeutic regimens have never been systematically studied.

Structured evidence on treatment-related safety of PD1/PD-L1 inhibitors would be necessary for clinicians in making clinical decisions. In this study, we carried out a systematic review and network meta-analysis (NMA) to compare the safety on trAEs and irAEs among different types of PD1/PD-L1 inhibitor-related therapeutic regimens simultaneously for cancer patients.

## Methods

### Study Design

This network meta-analysis was reported according to the Preferred Reporting Items for Systematic Reviews and Meta-Analyses (PRISMA) guidelines (25, 26). A priori established review protocol was followed when this study was conducted. The review protocol was not registered.

### Search Strategy and Selection Criteria

PubMed and Embase databases were systematically searched up to April 17th 2019 using the combinations of the following terms: (neoplasia OR malignancy OR melanoma) AND (nivolumab OR pembrolizumab OR lambrolizumab OR avelumab OR atezolizumab OR durvalumab OR “programmed cell death 1 receptor” OR “programmed cell death 1 ligand 1”) AND (random OR control OR placebo). The detailed search strategies are listed in Supplementary Table 1. There was no restriction on language or year of publication. We manually checked reference lists of related review articles and published trials to identify additional studies.

Studies were selected when they met the following inclusion criteria: (1) either PD-1 inhibitors (i.e., nivolumab, pembrolizumab) or PD-L1 inhibitors (i.e., atezolizumab, avelumab, durvalumab), alone or in combination with chemotherapy/placebo, were included in at least one of the treatment arms; (2) either PD-1/PD-L1 inhibitors, chemotherapy or placebo were included in the control arms; (3) treatment-related and/or immune-related adverse events were reported; (4) phase II or III RCTs. We excluded: (1) studies only in conference abstracts or posters form or presentations of ongoing trials; (2) study protocols, review, or commentary; (3) *in vitro* or animal studies; (4) studies which only involved quality-of-life outcomes or cost effectiveness analyses. Two investigators (HY, FH) selected the potentially eligible studies independently. The titles, abstracts, and full texts were evaluated sequentially.

### Data Extraction

Two independent investigators (HY and FH) extracted the data from all the eligible studies. The following information was extracted: trial name, line of treatment, study phase, blinding, median age (range), sex, tumor type, types, and dosage of drugs, number of patients in each randomization arm, median length of follow-up, number of patients in the safety dataset, number of patients with high-grade (grade 3–5) and all-grade (grade 1–5) treatment-related adverse events (trAEs), number of patients with high-grade and all-grade immune-related adverse events (irAEs). When more than one article reported the same outcome, we used the most updated data.

### Quality Assessment

The risk of bias was assessed independently by two authors (HY and FH) using Review Manager 5.3 software. The following domains were assessed: random sequence generation (selection bias), allocation concealment (selection bias), blinding of participants, and personnel (performance bias), blinding of outcome assessment (detection bias), incomplete outcome data (attrition bias), selective reporting (reporting bias), and other bias (27).

The Grading of Recommendations Assessment, Development, and Evaluation system (GRADE) approach was used to rate the quality of evidence (28). The four levels of evidence quality including high, moderate, low, and very low. The quality of evidence for each outcome was based on the fundamental study design and additional methodological factors.

### Outcome Measures

The term immune-related adverse events (irAEs) would not be appropriate to describe the chemotherapy toxicity. Therefore, treatment-related adverse events (trAEs) of both all-grade and high-grade were selected as the primary outcomes. The term trAEs was defined as all the adverse events that deemed to be treatment-related toxicities, based on the reporting of each original trial. The adverse events that were not described in the original trials as treatment-related were excluded. To avoid selective reporting, irAEs of both all-grade and high-grade were reported as the secondary outcomes. All the trAEs that deemed to be possible immune-related toxicities were irAEs. The all-grade and high-grade adverse events were defined as Grade 1–5 and Grade 3–5 adverse events, respectively, based on the National Cancer Institute Common Terminology Criteria for Adverse Events version 4.0.

### Data Synthesis and Statistical Analysis

Traditional pairwise meta-analyses were calculated using DerSimonian-Laird random-effects model since they were more conservative to deal with heterogeneity (29, 30). The summary effect sizes were presented as pooled odds ratio (OR) and 95% confidence intervals (CIs), with regard to the all-grade and high-grade trAEs and irAEs, respectively. A two-sided *p* < 0.05 or 95% CIs (excluding one) was regarded as statistically significant. We used the Cochrane *Q* statistic and quantified with *I*^2^ statistics to evaluate the heterogeneity among studies. An *I*^2^ value over 50% indicated substantial heterogeneity. Publication bias was examined using funnel plots (31).

NMA were performed based on Bayesian framework using a Markov Chain Monte Carlo (MCMC) simulation technique. The posterior distribution of all parameters was estimated using non-informative priors (32). The MCMC model was updated with 100,000 simulated draws after a burn-ins of 20,000 iterations and used a thinning interval of 10 for each chain. The adequacy of burn-in and convergence were assessed using the Brooks-Gelman-Rubin statistic (33). We reported the relative adverse effects of treatments as OR along with corresponding 95% credible intervals (Crls). Random-effects model was used since they generally showed better goodness of fit. Relative risks (RRs) and corresponding 95% CrIs were also calculated to estimate the incidence of trAEs and irAEs for each treatment (Incidence = 100 × assumed placebo risk × RR, the assumed placebo risk was generated by using traditional meta-analyses with random-effects model).

We calculated the posterior mean of the residual deviance to determine goodness of fit of the models. Ideally, each data point should contribute about one to the posterior mean of the residual deviance. It can be compared with the number of data points for model fit checking.

The median ranks and surface under the cumulative ranking curve (SUCRA) were estimated for all the arms and to obtain the hierarchy of safety. SUCRA was the percentage of drug safety on the adverse events that would be ranked first without uncertainty. When the drug safety is certain to be the best, the SUCRA value equals one. It equals zero when the safety is certain to be the worst (34).

Since consistency (defined as agreement) between direct and indirect results is the key to robust results, the presence of inconsistency was first evaluated by node splitting analysis in the entire network on particular comparisons (35, 36). Then we used the loop-specific approach to evaluate the presence of inconsistency in each loop (37). We calculated the values of two odds ratios (RoR) from direct and indirect evidence with 95% CI. *P*-value of <0.05 was regarded as significant inconsistency.

Meta-regression analyses were performed by adding pre-specified covariates (i.e., median age, percentage of male, line of treatment, tumor histology, whether double-blind design was used) to the network meta-analysis models. Sensitivity analyses were performed based on phase III trials only to evaluate the robustness of results. Subgroup analyses were conducted based on type of cancer (NSCLC and melanoma) and line of treatment (first-line, second, or higher line). The data analyses were conducted using STATA version 14.0 and WinBUGs version 1.4.3.

## Results

### Selection of Trials

**Figure 1:**
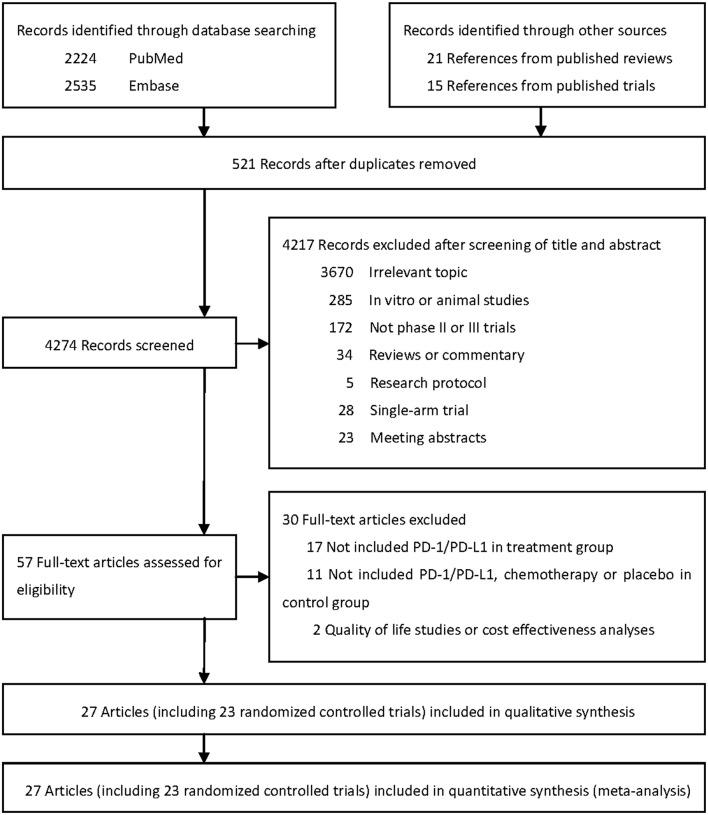
Literature search and selection.

Literature search initially identified 4,274 unduplicated articles, 4,217 of which were excluded after review of titles and abstracts. A total of 57 full-text articles were assessed for eligibility. Finally, 27 articles including 23 randomized controlled trials (RCTs) were included for quantitative synthesis (Figure 1) (1–6, 19–23, 38–53).

### Characteristics of Trials and Patients

**Figure 2:**
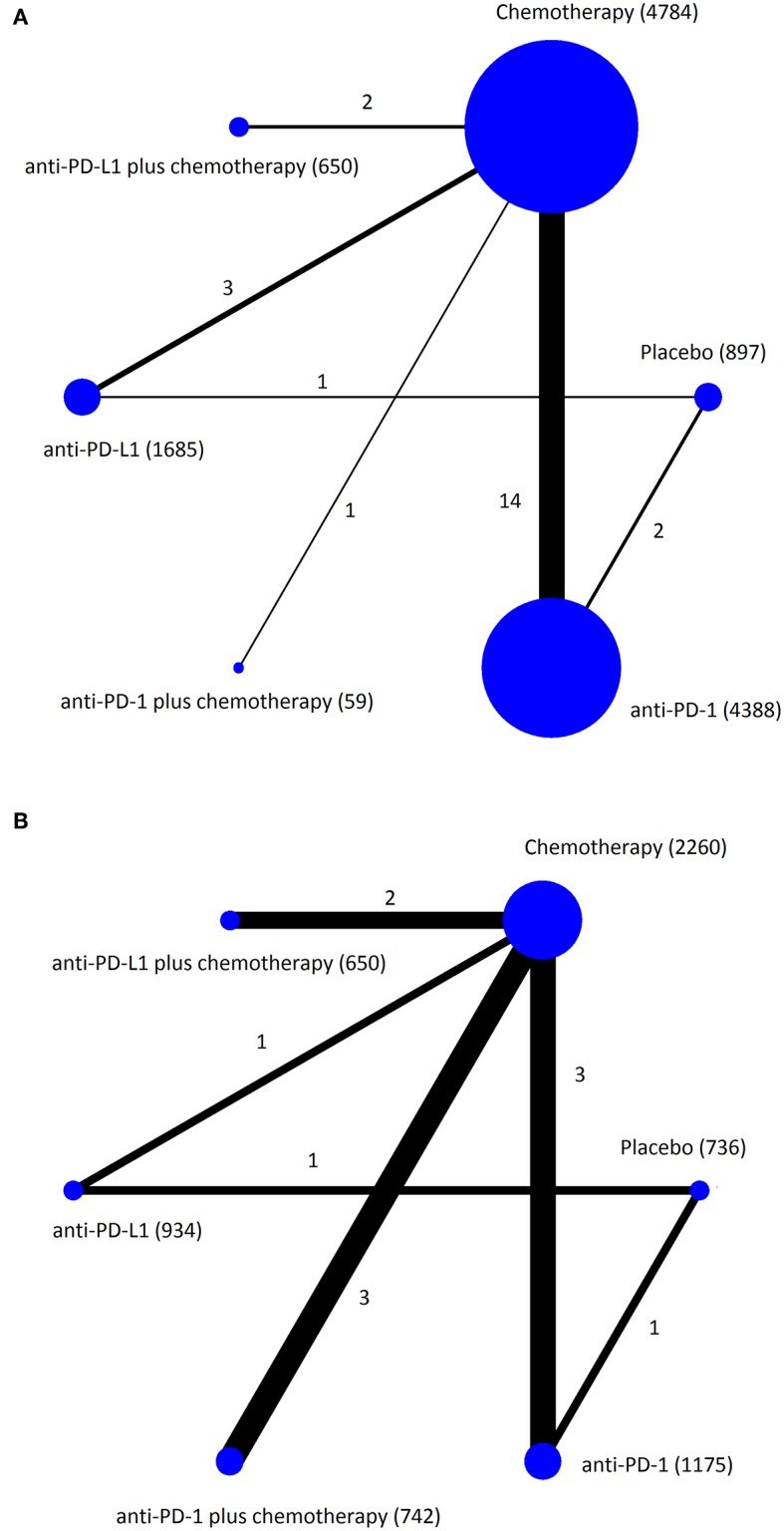
Network plots of eligible comparisons for the Bayesian network meta-analysis. The size of the nodes is proportional to the number of patients (in parentheses) randomized to receive the treatment. The width of the lines is proportional to the number of comparisons (beside the line) comparing the connected treatment (nodes). A total of 23 comparisons were analyzed for treatment-related adverse events **(A)**; a total of 11 comparisons were analyzed for immune-related adverse events **(B)**.

The 23 RCTs covered six treatment strategies and involved a total of 14,204 patients with cancer in the safety data set (Figure 2). Of the 23 included RCTs, 12 studied first-line treatment. Eight were phase III trials with double-blind design. Cancer types investigated included lung cancer (13), melanoma (4), gastric or gastro-esophageal junction cancer (2), urothelial carcinoma (2), breast cancer (1), head and neck carcinoma (1). PD-1 inhibitor (anti-PD-1) and PD-L1 inhibitor (anti-PD-L1) were evaluated in 17 and 6 trials, respectively. The baseline characteristics of included trials are listed in Table 1. The risk of bias summary for included trials is listed in Supplementary Table 2.

**Table 1:** Characteristics of included trials (27 articles including 23 randomized controlled trials).

**Trial name**	**Line of treatment**	**Study phase**	**Blinding**	**Median age (range)**	**Sex (male)**	**Tumor type**	**Treatment (number of patients in randomization)**	**Follow up months**	**Number of patients in safety dataset**	**Treatment-related adverse events**		**Immune-related adverse events**	
										**Grade 1–5**	**Grade 3–5**	**Grade 1–5**	**Grade 3–5**
CheckMate 017 (45)	Second-line	Phase 3	Open-label	63 (39–85)	208	Non-small-cell lung cancer	Nivolumab 3 mg/kg every 2 weeks (135)	Minimum 11.1	131	76	9	NA	NA
							Docetaxel 75 mg/m^2^ every 3 weeks (137)	Minimum 11.1	129	111	74	NA	NA
CheckMate 026 (49)	First-line	Phase 3	Open-label	64 (29–89)	332	Non-small-cell lung cancer	Nivolumab 3 mg/kg every 2 weeks (271)	Median 13.5	267	190	49	NA	NA
							Platinum doublet chemotherapy every 3 weeks (270)	Median 13.5	263	243	136	NA	NA
CheckMate 037 (20, 44)	Second-line	Phase 3	Open-label	60 (23–85)	261	Melanoma	Nivolumab 3 mg/kg every 2 weeks (272)	Median 8.4	268	206	37	NA	NA
							Dacarbazine 1,000 mg/m^2^ every 3 weeks or carboplatin AUC = 6 plus paclitaxel 175 mg/m^2^ every 3 weeks (133)	Median 8.4	102	84	35	NA	NA
CheckMate 057 (46)	Second-line	Phase 3	Open-label	62 (21–85)	319	Non-small-cell lung cancer	Nivolumab 3 mg/kg every 2 weeks (292)	Minimum 13.2	287	199	30	NA	NA
							Docetaxel 75 mg/m^2^ every 3 weeks (290)	Minimum 13.2	268	236	144	NA	NA
CheckMate 066 (2, 23)	First-line	Phase 3	Double-blind	65 (18–87)	246	Melanoma	Nivolumab 3 mg/kg every 2 weeks (210)	Median 8.9	206	160	31	NA	NA
							Dacarbazine 1,000 mg/m^2^ every 3 weeks (208)	Median 6.8	205	159	36	NA	NA
CheckMate 227 (47)	First-line	Phase 3	Open-label	64 (29–87)	NA	Non-small-cell lung cancer	Nivolumab 240 mg every 2 weeks (573)	Minimum 11.2	391	251	76	NA	NA
							Platinum doublet chemotherapy based on tumor histologic type every 3 weeks (583)	Minimum 11.2	570	460	216	NA	NA
IMpassion130 (6)	First-line	Phase 3	Double-blind	55 (20–86)	0	Breast cancer	Atezolizumab 840 mg on days 1 and 15 plus nab-paclitaxel 100 mg/m^2^ on days 1, 8, and 15 every 4 weeks (451)	Median 12.9	452	436	182	259	34
							NAb-paclitaxel 100 mg/m^2^ on days 1, 8, and 15 every 4 weeks (451)	Median 12.9	438	410	133	183	19
IMpower133 (5)	First-line	Phase 3	Double-blind	64 (26–90)	261	Small-cell lung cancer	Carboplatin AUC = 5 every 3 weeks and etoposide 100 mg/m^2^ with atezolizumab 1,200 mg (201)	Median 13.9	198	188	115	79	NA
							Carboplatin AUC = 5 every 3 weeks and etoposide 100 mg/m^2^ (202)	Median 13.9	196	181	113	48	NA
IMvigor211 (52)	First-line	Phase 3	Open-label	67 (31–88)	718	Urothelial carcinoma	Atezolizumab 1,200 mg every 3 weeks (467)	Median 17.3	459	319	95	139	37
							Vinflunine 320 mg/m^2^, paclitaxel 175 mg/m^2^, or docetaxel 75 mg/m^2^ every 3 weeks (464)	Median 17.3	443	395	198	98	14
KEYNOTE-002 (1, 39)	Second-line or more	Phase 2	Open-label	62 (15–89)	327	Melanoma	Pembrolizumab 2 mg/kg every 3 weeks (180)	Median 10	178	125	24	NA	NA
							Pembrolizumab 10 mg/kg every 3 weeks (181)	Median 10	179	136	30	NA	NA
							Paclitaxel plus carboplatin, paclitaxel, carboplatin, dacarbazine, or oral temozolomide (179)	Median 10	171	138	45	NA	NA
KEYNOTE-010 (40)	Second-line or more	Phase 2/3	Open-label	63 (56–69)	634	Non-small-cell lung cancer	Pembrolizumab 2 mg/kg every 3 weeks (345)	Median 13.1	339	215	43	NA	NA
							Pembrolizumab 10 mg/kg every 3 weeks (346)	Median 13.1	343	226	55	NA	NA
							Docetaxel 75 mg/m^2^ every 3 weeks (343)	Median 13.1	309	251	109	NA	NA
KEYNOTE-021 (50)	First-line	Phase 3	Open-label	63 (54–70)	48	Non-small-cell lung cancer	Pembrolizumab 200 mg every 3 weeks plus pemetrexed 500 mg/m^2^ and carboplatin AUC = 5 every 3 weeks followed by pembrolizumab for 24 months and optional indefinite pemetrexed maintenance therapy (60)	Median 10.6	59	55	23	13	2
							Pemetrexed 500 mg/m^2^ and carboplatin AUC = 5 followed by optional indefinite pemetrexed maintenance therapy (63)	Median 10.6	62	56	16	7	1
KEYNOTE-024 (22)	First-line	Phase 3	Open-label	65 (33–90)	187	Non-small-cell lung cancer	Pembrolizumab 200 mg every 3 weeks (154)	Median 11.2	154	113	41	45	15
							Carboplatin plus pemetrexed, cisplatin plus pemetrexed, carboplatin plus gemcitabine, cisplatin plus gemcitabine, or carboplatin plus paclitaxel (151)	Median 11.2	150	135	80	7	1
KEYNOTE-040 (3)	First-line	Phase 3	Open-label	60 (54–66)	412	Head and neck carcinoma	Pembrolizumab 200 mg every 3 weeks (247)	Median 7.5	246	155	33	63	11
							Methotrexate 40 mg/m^2^ or docetaxel 75 mg/m^2^ every 3 weeks or cetuximab 250 mg/m^2^ per week following a loading dose of 400 mg/m^2^ (248)	Median 7.1	234	196	85	28	11
KEYNOTE-045 (43)	Second-line	Phase 3	Open-label	66 (26–88)	402	Urothelial carcinoma	Pembrolizumab 200 mg every 3 weeks (270)	Median 14.1	266	162	40	45	12
							Paclitaxel 175 mg/m^2^ every 3 weeks or docetaxel 75 mg/m^2^ or vinflunine 320 mg/m^2^ every 3 weeks (272)	Median 14.1	255	230	126	19	4
KEYNOTE-054 (53)	Second-line or more	Phase 3	Double-blind	54 (19–88)	628	Melanoma	Pembrolizumab 200 mg every 3 weeks (514)	Median 15	509	396	75	190	36
							Placebo (505)	Median 15	502	332	17	45	3
KEYNOTE-061 (38)	First-line	Phase 3	Open-label	61 (53–70)	410	Gastric or gastro-esophageal junction cancer	Pembrolizumab 200 mg every 3 weeks (296)	Median 8.5	294	155	42	NA	NA
							Paclitaxel 80 mg/m^2^ every 4 weeks (296)	Median 7.5	276	232	96	NA	NA
KEYNOTE-189 (41)	First-line	Phase 3	Double-blind	64 (34–84)	363	Non-small-cell lung cancer	Pembrolizumab 200 mg every 3 weeks plus pemetrexed and a platinum-based drug every 3 weeks followed by pemetrexed maintenance therapy (410)	Median 10.5	405	NA	NA	92	36
							Pemetrexed and a platinum-based drug every 3 weeks followed by pemetrexed maintenance therapy (206)	Median 10.5	202	NA	NA	24	9
KEYNOTE-407 (42)	First-line	Phase 3	Double-blind	65 (29–88)	455	Non-small-cell lung cancer	Pembrolizumab 200 mg plus carboplatin 6 mg/m^2^ and paclitaxel 200 mg/m^2^ or nab-paclitaxel 100 mg/m^2^ every 3 weeks (278)	Median 7.8	278	NA	NA	80	30
							Carboplatin 6 mg/m^2^ and paclitaxel 200 mg/m^2^ or nab-paclitaxel 100 mg/m^2^ every 3 weeks (281)	Median 7.8	280	NA	NA	24	9
OAK (51)	Second-line or more	Phase 3	Open-label	63 (33–85)	747	Non-small-cell lung cancer	Atezolizumab 1,200 mg every 3 weeks (613)	Median 21	609	390	90	NA	NA
							Docetaxel 75 mg/m^2^ every 3 weeks (612)	Median 21	578	496	248	NA	NA
ONO-4538-12, ATTRACTION-2 (48)	Second-line or more	Phase 3	Double-blind	62 (53–69)	348	Gastric or gastro-esophageal junction cancer	Nivolumab 3 mg/kg every 2 weeks (330)	Median 8.9	330	141	39	NA	NA
							Placebo (163)	Median 8.6	161	43	9	NA	NA
PACIFIC study (4, 21)	Second-line or more	Phase 3	Double-blind	64 (23–90)	500	Non-small-cell lung cancer	Durvalumab 10 mg/kg every 2 weeks (476)	Median 25.2	475	322	63	115	20
							Placebo (237)	Median 25.2	234	125	13	19	9
POPLAR Study (19)	Second-line or more	Phase 2	Open-label	62 (36–84)	169	Non-small-cell lung cancer	Atezolizumab 1,200 mg every 3 weeks (144)	Median 14.8	142	95	17	NA	NA
							Docetaxel 75 mg/m^2^ every 3 weeks (143)	Median 15.7	135	119	55	NA	NA

### Treatment-Related Adverse Events (trAEs)

Results of pairwise meta-analyses showed that anti-PD-L1 plus chemotherapy was associated with increased all-grade trAEs compared with using chemotherapy alone (OR = 1.74, 95% CI: 1.06–2.88), with high quality of evidence. However, anti-PD-L1 monotherapy was associated with a significant reduction in trAEs compared with chemotherapy (all-grade: OR = 0.29, 95% CI: 0.23–0.35; high-grade: OR = 0.26, 95% CI: 0.20–0.34), with moderate quality of evidence. Compared with placebo, both anti-PD-1 and anti-PD-L1 were associated with significantly increased high-grade trAEs, with moderate to high quality of evidence (Supplementary Tables 3, 4).

Median ranks on treatment-related safety from high to low were: placebo, anti-PD-L1, anti-PD-1, chemotherapy, anti-PD-1 plus chemotherapy, anti-PD-L1 plus chemotherapy for all-grade trAEs; placebo, anti-PD-L1, anti-PD-1, chemotherapy, anti-PD-L1 plus chemotherapy, anti-PD-1 plus chemotherapy for high-grade trAEs (Supplementary Table 5). Pooled all-grade and high-grade incidences of trAEs were, respectively, 48.9 and 4.3% for placebo, 60.4 and 6.4% for anti-PD-L1, 65.7 and 6.6% for anti-PD-1, 82.8 and 8.0% for chemotherapy, 87.4 and 8.2% for anti-PD-1 plus chemotherapy, 88.6 and 8.1% for anti-PD-L1 plus chemotherapy.

**Figure 3:**
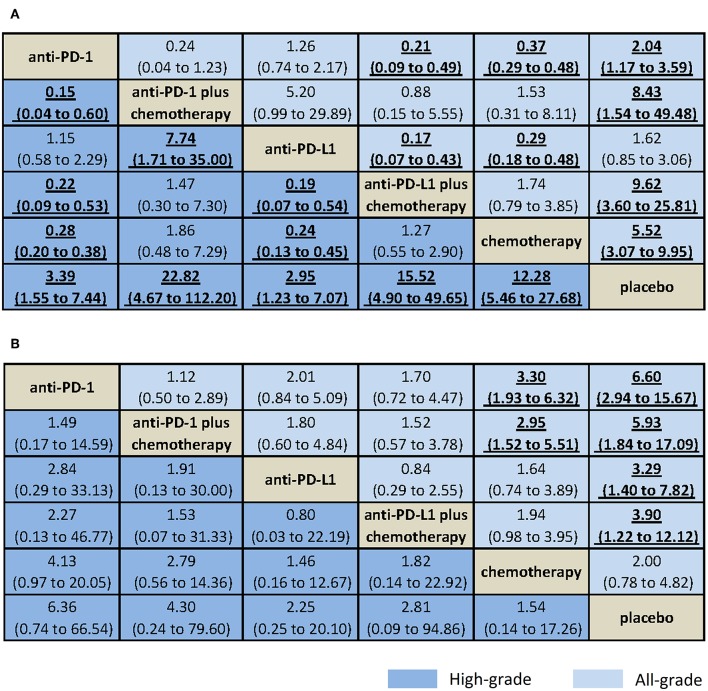
Bayesian network meta-analysis of treatment-related and immune-related adverse events. Comparisons should be read from the top treatment to the bottom treatment. Bold underline cells are significant. Results represent pooled odds ratios and 95% credible intervals for treatment-related adverse events **(A)** and immune-related adverse events **(B)**. Odds ratio >1 favors the bottom treatment.

**Figure 4:**
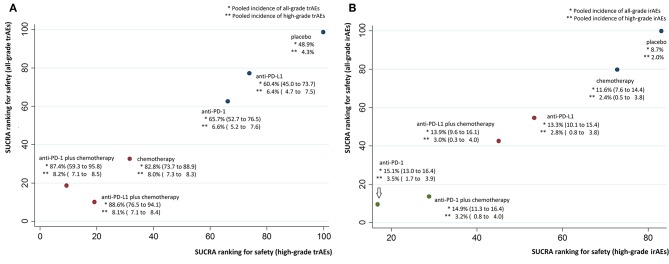
Cluster SUCRA ranking plots and incidence. **(A)** Cluster SUCRA ranking plot for safety on all-grade and high-grade trAEs (x-axis: SUCRA ranking for safety on high-grade trAEs; y-axis: SUCRA ranking for safety on all-grade trAEs) and incidence. **(B)** Cluster SUCRA ranking plot for safety on all-grade and high-grade irAEs (x-axis: SUCRA ranking for safety on high-grade irAEs; y-axis: SUCRA ranking for safety on all-grade irAEs) and incidence. For trAEs and irAEs, higher SUCRA ranking = safer treatment with lower risk of adverse events. Numbers in parenthesis represent 95% CIs derived by replacing the relative risks in the calculation with the lower and upper limits of their respective 95% CrIs. irAEs, immune-related adverse events; trAEs, treatment-related adverse events.

Results from NMA and SUCRA suggested that, compared with placebo, anti-PD-L1 monotherapy did not increase trAEs significantly (all-grade: OR = 1.62, 95% CrI: 0.85–3.06, SUCRA = 77.2%; high-grade: OR = 2.95, 95% CrI: 1.23–7.07, SUCRA = 73.8%). However, anti-PD-L1 plus chemotherapy had the worst safety on all-grade trAEs (all-grade: OR = 9.62, 95% CrI: 3.60–25.81, SUCRA = 10.1%. Anti-PD-1 plus chemotherapy had the worst safety on high-grade trAEs (OR = 15.52, 95% CrI: 4.90–49.65, SUCRA = 9.3%), with moderate to high quality of evidence (Figures 3A, 4A, Supplementary Table 4).

When comparative safety was assessed, anti-PD-L1 and anti-PD-1 were superior to all other treatments (not include placebo) for the safety on high-grade trAEs. Anti-PD-L1 and anti-PD-1 were comparable with each other for trAEs (all-grade: OR = 1.26, 95% CrI: 0.74–2.17; high-grade: OR = 1.15, 95% CrI: 0.58–2.29), both with high quality of evidence. When combined with chemotherapy, anti-PD-L1 and anti-PD-1 were also comparable with each other for trAEs (all-grade: OR = 0.88, 95% CrI: 0.15–5.55; high-grade: OR = 1.47, 95% CrI: 0.30–7.30), with high quality of evidence (Figure 3A, Supplementary Table 4).

### Immune-Related Adverse Events (irAEs)

Results of pairwise meta-analyses showed that both anti-PD-1 and anti-PD-L1 and their combination with chemotherapy were associated with increased all-grade irAEs compared with using chemotherapy alone, with low to high quality of evidence. Compared with placebo, anti-PD-1 was associated with increased high-grade irAEs (OR = 12.66, 95% CI: 3.87–41.38, with moderate quality of evidence), whereas anti-PD-L1 was not (OR = 1.10, 95% CI: 0.49–2.45, with moderate quality of evidence; Supplementary Tables 3, 4).

Median ranks on immune-related safety from high to low were: placebo, chemotherapy, anti-PD-L1, anti-PD-L1 plus chemotherapy, anti-PD-1 plus chemotherapy, anti-PD-1 for both all-grade and high-grade irAEs (Supplementary Table 5). Pooled all-grade and high-grade incidences of irAEs were, respectively, 11.6 and 2.4% for chemotherapy, 13.3 and 2.8% for anti-PD-L1, 13.9 and 3.0% for anti-PD-L1 plus chemotherapy, 14.9 and 3.2% for anti-PD-1 plus chemotherapy, 15.1 and 3.5% for anti-PD-1.

Results from NMA suggested that, compared with placebo, anti-PD-1 monotherapy increased all-grade irAEs significantly (all-grade: OR = 6.60, 95% CrI: 2.94–15.67, SUCRA = 9.5%; high-grade: OR = 6.36, 95% CrI: 0.74–66.54, SUCRA = 16.8%), with moderate to high quality of evidence (Figures 3B, 4B, Supplementary Table 4).

When comparative safety was assessed, compared with chemotherapy, anti-PD-1 was associated with increased all-grade irAEs (OR = 3.30, 95% CrI: 1.93–6.32), whereas anti-PD-L1 was not (OR = 1.64, 95% CrI: 0.74–3.89). However, both were with low quality of evidence. Compared with chemotherapy, anti-PD-1 plus chemotherapy was associated with increased all-grade irAEs (OR = 2.95, 95% CrI: 1.52–5.51, with low quality of evidence), whereas anti-PD-L1 plus chemotherapy was not (OR = 1.94, 95% CrI: 0.98–3.95, with high quality of evidence). Anti-PD-L1 and anti-PD-1 were comparable with each other for irAEs (all-grade: OR = 2.01, 95% CrI: 0.84–5.09; high-grade: OR = 2.84, 95% CrI: 0.29–33.13), with high quality of evidence. When combined with chemotherapy, anti-PD-L1 and anti-PD-1 were also comparable with each other for irAEs (all-grade: OR = 1.52, 95% CrI: 0.57–3.78; high-grade: OR = 1.53, 95% CrI: 0.07–31.33), with high quality of evidence (Figure 3B, Supplementary Table 4).

### Model Fit, Inconsistence Check, and Quality of Evidence

The model fit was evaluated using the posterior mean of the residual deviance, which was 42 and 43 for trAEs of all-grade and high-grade, respectively, and was 20 and 19 for irAEs of all-grade and high-grade, respectively. The model's overall fit was relatively satisfactory. Inconsistence check showed that neither node splitting analysis nor loop-specific approach showed significant inconsistency between direct and indirect results, which indicated robust results (Supplementary Tables 6, 7). Supplementary Table 4 summarized the quality of evidence using GRADE framework for the outcomes. Overall, there was no inconsistency of results, indirectness or publication bias. In several direct comparisons, study limitations existed since lack of blinding. In NMA, three comparisons had serious imprecision in summary estimate.

### Reporting Bias, Sensitivity Analyses, and Meta-Regression

Supplementary Figure 1 presents the adjusted funnel plot for the network. The funnel plots of all-grade and high-grade of trAEs and irAEs did not show asymmetry, suggesting no potential risk of reporting bias. Sensitivity analyses based on phase III trials did not indicate any major influence on the outcomes (Supplementary Figures 2, 3). Meta-regression analyses did not reveal any pre-specified factors that influenced the estimated effects significantly (Supplementary Table 8).

### Subgroup Analyses

Among patients with NSCLC, median ranks on treatment-related safety from high to low were: placebo, anti-PD-L1, anti-PD-1, chemotherapy, anti-PD-1 plus chemotherapy. Median ranks on immune-related safety from high to low were: chemotherapy, anti-PD-1 plus chemotherapy, anti-PD-1 (Supplementary Tables 9, 10). The pooled incidence of trAEs was highest for anti-PD-1 plus chemotherapy (all-grade: 97.0%; high-grade: 10.7%; Supplementary Table 11). Compared with chemotherapy, both anti-PD-1 and anti-PD-L1 were associated with decreased trAEs, whereas anti-PD-1 increased irAEs significantly (Supplementary Table 12).

Among patients with melanoma, median ranks on treatment-related safety from high to low were: placebo, anti-PD-1, chemotherapy (Supplementary Tables 9, 10). For anti-PD-1, the pooled incidence was 85.0 and 5.6% for all-grade and high-grade trAEs, respectively (Supplementary Table 11).

Among patients with first-line treatment, median ranks on treatment-related safety high to low were anti-PD-L1, anti-PD-1, chemotherapy, anti-PD-1 plus chemotherapy, anti-PD-L1 plus chemotherapy. Median ranks on immune-related safety from high to low were: chemotherapy, anti-PD-L1, anti-PD-L1 plus chemotherapy, anti-PD-1 plus chemotherapy, anti-PD-1 (Supplementary Tables 9, 10). The pooled incidence of trAEs was lowest for anti-PD-L1 (all-grade: 37.8%; high-grade 19.0%), whereas anti-PD-1 had the highest irAEs incidence (all-grade: 27.6%; high-grade: 4.5%; Supplementary Table 11). Compared with chemotherapy, anti-PD-1 was associated with decreased trAEs of both all-grade and high-grade. However, anti-PD-1 was associated with increased all-grade irAEs compared with chemotherapy (Supplementary Table 12).

Among patients with second or higher line of treatment, median ranks on treatment-related safety from high to low were: placebo, anti-PD-L1, anti-PD-1, chemotherapy (Supplementary Tables 9, 10). The pooled incidence of all-grade trAEs was 65.6 and 60.5% for anti-PD-1 and anti-PD-L1, respectively (Supplementary Table 11). Compared with chemotherapy, both anti-PD-1 and anti-PD-L1 decreased trAEs significantly (Supplementary Table 12).

## Discussion

### Summary of Key Findings

This NMA compares all anti-PD1/PD-L1-related therapeutic regimens for cancer patients. There are three key findings. First, anti-PD-L1 monotherapy was ranked the best for safety on trAEs. However, when combined with chemotherapy, both anti-PD-L1 and anti-PD-1 were ranked the worst. Second, anti-PD-L1 monotherapy had the lowest irAEs incidence, whereas anti-PD-1 monotherapy had the highest. However, anti-PD-L1 did not decrease irAEs significantly compared with anti-PD-1. Third, both anti-PD-L1 and anti-PD-1 monotherapy decreased high-grade trAEs significantly compared with using chemotherapy alone or combining chemotherapy with anti-PD-1/anti-PD-L1.

### Comparison With Other Studies

One study showed that combinations of conventional therapy with immune checkpoint inhibitors (ICIs) would be associated with increased trAEs compared with taking one ICI drug (17). Another study showed that anti-PD-1 would slightly increase trAEs compared with anti-PD-L1 (54). This NMA agreed with these previous findings. In addition, we calculated the safety ranking on trAEs. We found that anti-PD-L1 monotherapy had the lowest incidences on both trAEs and irAEs among anti-PD-1/anti-PD-L1-related therapeutic regimens.

Xu et al. reported all-grade trAEs incidences of 73.5 and 66.4% for anti-PD-1 and anti-PD-L1, respectively (17). In our study, lower pooled incidence of trAEs was obtained, which was 65.7 and 60.4% for anti-PD-1 and anti-PD-L1, respectively. Xu et al. used all adverse events if trAE was not available (17), whereas we used trAEs data only. Thus, the overall incidence would not be overestimated. Pillai et al. compared irAEs between anti-PD-1 and anti-PD-L1 among patients with NSCLC (54). Both would lead to 3.0% high-grade irAEs incidence. Our study included patients with all types of cancer who showed an incidence of 3.5 and 2.8% for anti-PD-1 and anti-PD-L1, respectively. In addition, we found that anti-PD-1 monotherapy would increase irAEs compared with chemotherapy, whereas anti-PD-L1 monotherapy would not. Compared with placebo, all the anti-PD-1/anti-PD-L1-related therapeutic regimens would increase all-grade irAEs, but not high-grade irAEs.

### Strength and Limitations of Study

This NMA fills a crucial knowledge gap regarding the comparative risks of both trAEs and irAEs among PD-1/PD-L1 inhibitors and their combination with chemotherapy. However, four limitations should be noted. First, each individual drug would have its distinct toxicity profile. However, data analyses in this study were performed by classifying drugs according to the mechanism of action rather than analyzing the drugs separately due to a limited number of trials available at this moment so as to avoid yielding very sparse networks. Combining different drugs of the same class within a single category of targeted agent may introduce heterogeneity in the NMA. Nevertheless, the random-effects model was used and the model's overall fit was satisfactory. More original trials are still awaited to explore more about the toxicity profiles for each drug. Second, indirect comparisons from NMA may suffer bias through confounding by study-level characteristics. Results from indirect comparisons should be interpreted with caution as direct comparison is lacking. However, we included RCTs only in this NMA, thus the trial populations and study characteristics were comparable to the target population. Potential confounding factors were further evaluated using meta-regression analyses, which had no major influence on the results. Third, node split analysis showed a *P*-value of 0.08 in four comparisons of high-grade irAEs. Despite there being no statistical inconsistency, further evidence with outstanding consistency is still needed when more trials are available for these comparisons. Last but not least, immune-related diseases are usually late onset, thus the incidence of irAEs may be underestimated due to limited follow-up time of the included trials.

### Clinical and Research Implications

This current study sheds light on the important clinical issue about the comparative safety on both trAEs and irAEs from anti-PD-L1/anti-PD-1-related treatment. Our results demonstrated that anti-PD-L1 was well-tolerated for all cancer patients. A previous study showed that different tumor histologies may have different irAEs profiles (55). In our study, results of meta-regression showed that tumor histology did not influence the estimated effects.

Among patients with NSCLC, Xu et al. stated that nivolumab (anti-PD-1) rather than anti-PD-L1 would be ranked the best for safety on trAEs. However, this finding could be considered with caution in clinical practice. First, PACIFIC study was not included in their analysis (4, 21). We found that anti-PD-L1 monotherapy was still ranked the best for all-grade trAEs if the PACIFIC study was included among NSCLC. Second, we found that among the nivolumab trials available, none of them reported all-grade or high-grade irAEs. It will be interesting to learn how the conclusion changes with more irAEs data being reported. The immune-related safety needs to be investigated further for nivolumab.

There are two research implications. First, the irAEs associated with anti-PD-1/anti-PD-L1 may be distinct. For example, anti-PD-1 agents may have higher risk of immune-related pneumonitis, whereas, anti-PD-L1 agents may have higher hypothyroidism risk (56). More studies should be conducted in the future base on organ specific immune-related adverse events, especially focusing on colitis, hepatitis (aspartate aminotransferase), pneumonitis, hypothyroidism, and rash. Second, in real-world clinical practice, it is still challenging to discern irAEs since adverse events, such like pneumonitis and colitis, may be caused by non-immune-related reactions. In the future, it is important to publish standardized method to specify the clinical criteria for irAEs.

## Conclusion

Anti-PD-L1 monotherapy was ranked the best for safety on trAEs, whereas anti-PD-1/anti-PD-L1 plus chemotherapy was ranked the worst. Among the anti-PD-1/PD-L1-related therapeutic regimens, anti-PD-L1 had the best irAEs safety, whereas anti-PD-1 had the worst. Anti-PD-1 did not increase trAEs or irAEs significantly compared with anti-PD-L1. Awareness surrounding the comparative safety of trAEs and irAEs from anti-PD-1/PD-L1-related therapeutic regimens could promote further appropriate utilization of these agents in clinical practice.

## Data Availability Statement

All data generated or analyzed during this study are included in this published article.

## Author Contributions

YH, WX, and JD: study conception and paper writing. YH and WX: study design and discussion of the findings. YH and HF: data extraction and elaboration. YH, WX, and HF: data analysis and interpretation. YH, WX, HF, and JD: all coauthors have read and approved the manuscript in its present form, agreed to be personally accountable for the author's own contributions and to ensure that questions related to the accuracy or integrity of any part of the work, even ones in which the author was not personally involved, are appropriately investigated, resolved, and the resolution documented in the literature.

### Conflict of Interest

The authors declare that the research was conducted in the absence of any commercial or financial relationships that could be construed as a potential conflict of interest.

## Supplementary Material

Supplementary Figure 1 Comparison—adjusted funnel plot for the network. Adjusted funnel plot for the network for all-grade treatment-related adverse events **(A)**, high-grade treatment-related adverse events **(B)**, all-grade immune-related adverse events **(C)**, and high-grade immune-related adverse events **(D)**. The red line represents the null hypothesis that the study-specific effect sizes do not differ from the respective comparison-specific pooled effect estimates. The blue line is the regression line. Different colors correspond to different comparisons. 01, Placebo; 02, Chemotherapy; 03, anti-PD-L1 plus chemotherapy; 04, anti-PD-L1; 05, anti-PD-1 plus chemotherapy; 06, anti-PD-1 inhibitors.

Supplementary Figure 2 Bayesian network meta-analysis of treatment-related and immune-related adverse events (sensitivity analysis). Comparisons should be read from the top treatment to the bottom treatment. Bold underline cells are significant. Results represent pooled odds ratios and 95% credible intervals for treatment-related adverse events **(A)** and immune-related adverse events **(B)**. Odds ratio >1 favors the bottom treatment.

Supplementary Figure 3 Cluster SUCRA ranking plots (sensitivity analysis). **(A)** Cluster SUCRA ranking plot for safety on all-grade and high-grade trAEs (x-axis: SUCRA ranking for safety on high-grade trAEs; y-axis: SUCRA ranking for safety on all-grade trAEs). **(B)** Cluster SUCRA ranking plot for safety on all-grade and high-grade irAEs (x-axis: SUCRA ranking for safety on high-grade irAEs; y-axis: SUCRA ranking for safety on all-grade irAEs). For trAEs and irAEs, higher SUCRA ranking = safer treatment with lower risk of adverse events. irAEs, immune-related adverse events; trAEs, treatment-related adverse events.

Supplementary Table 1 Search strategies.

Supplementary Table 2 Risk of bias summary.

Supplementary Table 3 Results of traditional pairwise meta-analyses for different outcomes.

Supplementary Table 4 Evaluation of the quality of evidence using GRADE framework for outcomes.

Supplementary Table 5 Median ranks on safety (rank 1–6 on each scale) and 95% CrIs.

Supplementary Table 6 Nodesplit analysis of network meta-analysis for treatment-related adverse events and immune-related adverse events.

Supplementary Table 7 Evaluation of inconsistency using loop-specific heterogeneity estimates.

Supplementary Table 8 Meta-regression for treatment-related adverse events and immune-related adverse events.

Supplementary Table 9 Subgroup analysis: median ranks on safety (rank 1–6 on each scale) and 95% CrIs.

Supplementary Table 10 SUCRA rankings in the subgroup analysis based on type of cancer and line of treatment.

Supplementary Table 11 Pooled incidence along with corresponding 95% credible intervals of treatment based on type of cancer and line of treatment.

Supplementary Table 12 Bayesian network meta-analysis based on type of cancer and line of treatment.

## Abbreviations

AEs: adverse events
CIs: confidence intervals
CrIs: credible intervals
FDA: Food and Drug Administration
ICIs: immune checkpoint inhibitors
irAEs: immune-related adverse events
NA: not available
NE: not estimable
NSCLC: non-small cell lung cancer
OR: odds ratio
PD-1: programmed cell death 1
PD-L1: programmed cell death-ligand 1
trAEs: treatment-related adverse events.
