# Supplementary figures and images for: Comparative Safety of PD-1/PD-L1 Inhibitors for Cancer Patients: Systematic Review and Network Meta-Analysis
Source: Front Oncol. 2019 Oct 1;9:972. doi: 10.3389/fonc.2019.00972 (PMC6779807; doi:10.3389/fonc.2019.00972)

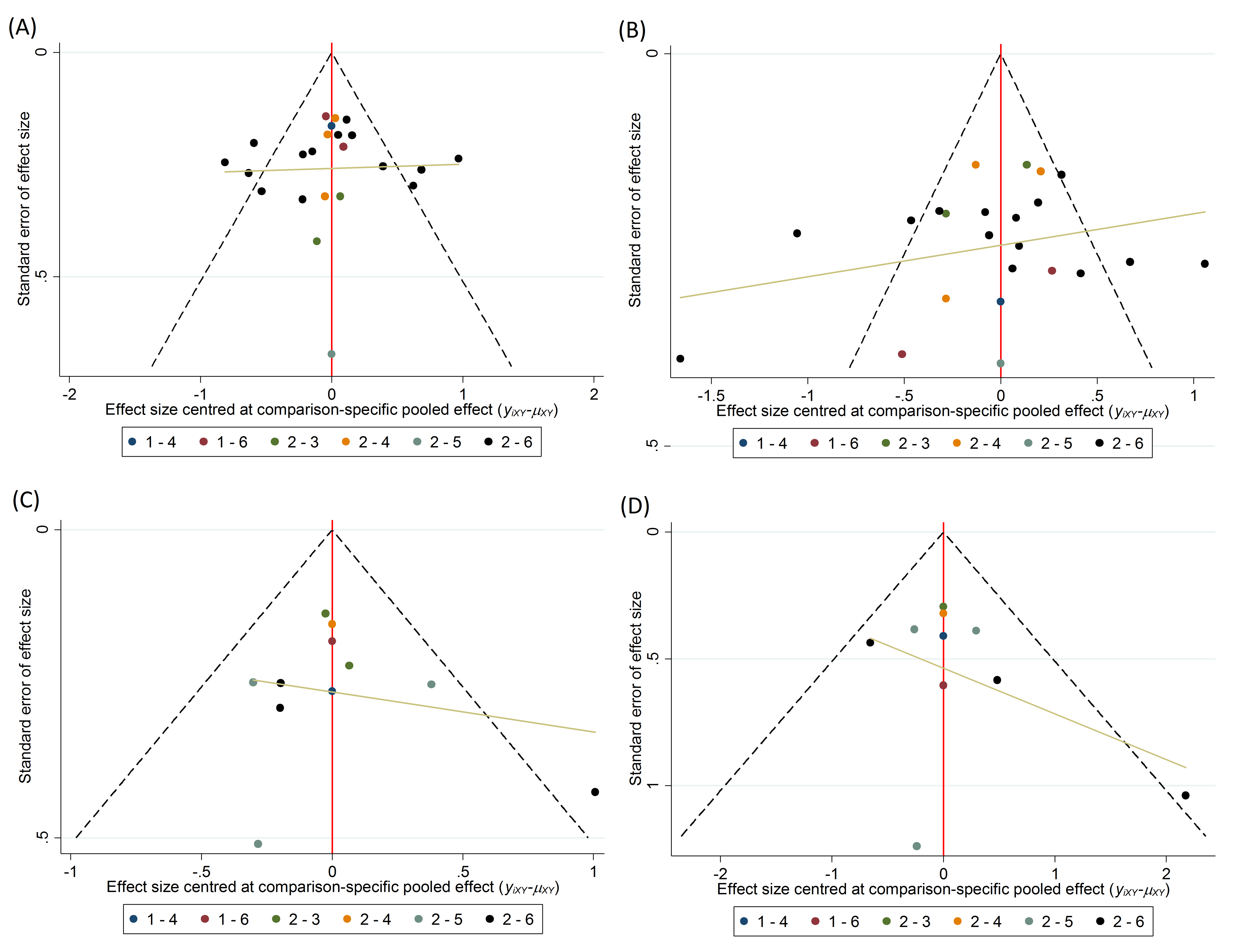

Supplement: Supplementary Figure 1 — Comparison—adjusted funnel plot for the network. Adjusted funnel plot for the network for all-grade treatment-related adverse events (A), high-grade treatment-related adverse events (B), all-grade immune-related adverse events (C), and high-grade immune-related adverse events (D). The red line represents the null hypothesis that the study-specific effect sizes do not differ from the respective comparison-specific pooled effect estimates. The blue line is the regression line. Different colors correspond to different comparisons. 01, Placebo; 02, Chemotherapy; 03, anti-PD-L1 plus chemotherapy; 04, anti-PD-L1; 05, anti-PD-1 plus chemotherapy; 06, anti-PD-1 inhibitors. [file Image_1.TIF]

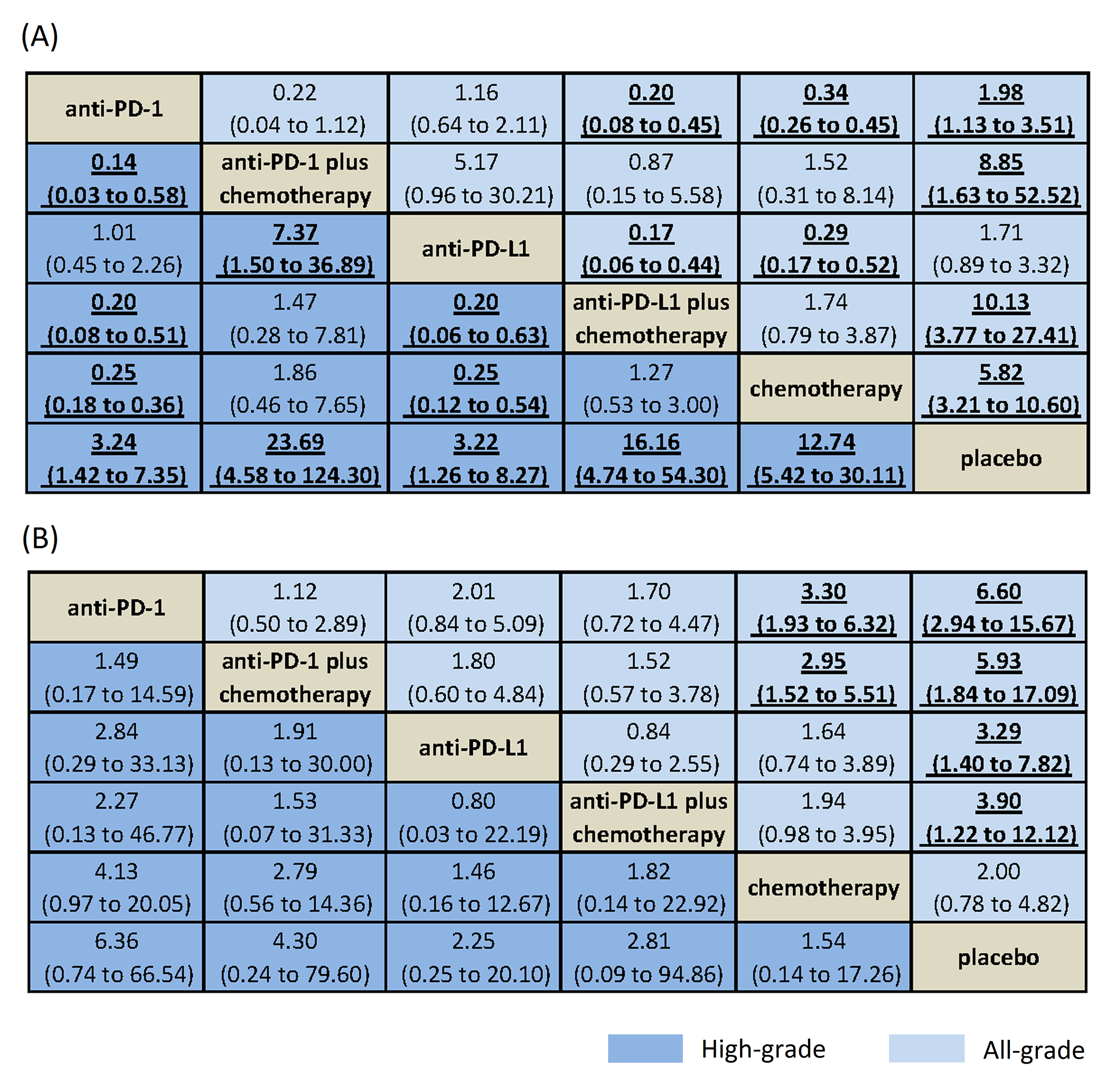

Supplement: Supplementary Figure 2 — Bayesian network meta-analysis of treatment-related and immune-related adverse events (sensitivity analysis). Comparisons should be read from the top treatment to the bottom treatment. Bold underline cells are significant. Results represent pooled odds ratios and 95% credible intervals for treatment-related adverse events (A) and immune-related adverse events (B). Odds ratio >1 favors the bottom treatment. [file Image_2.TIFF]

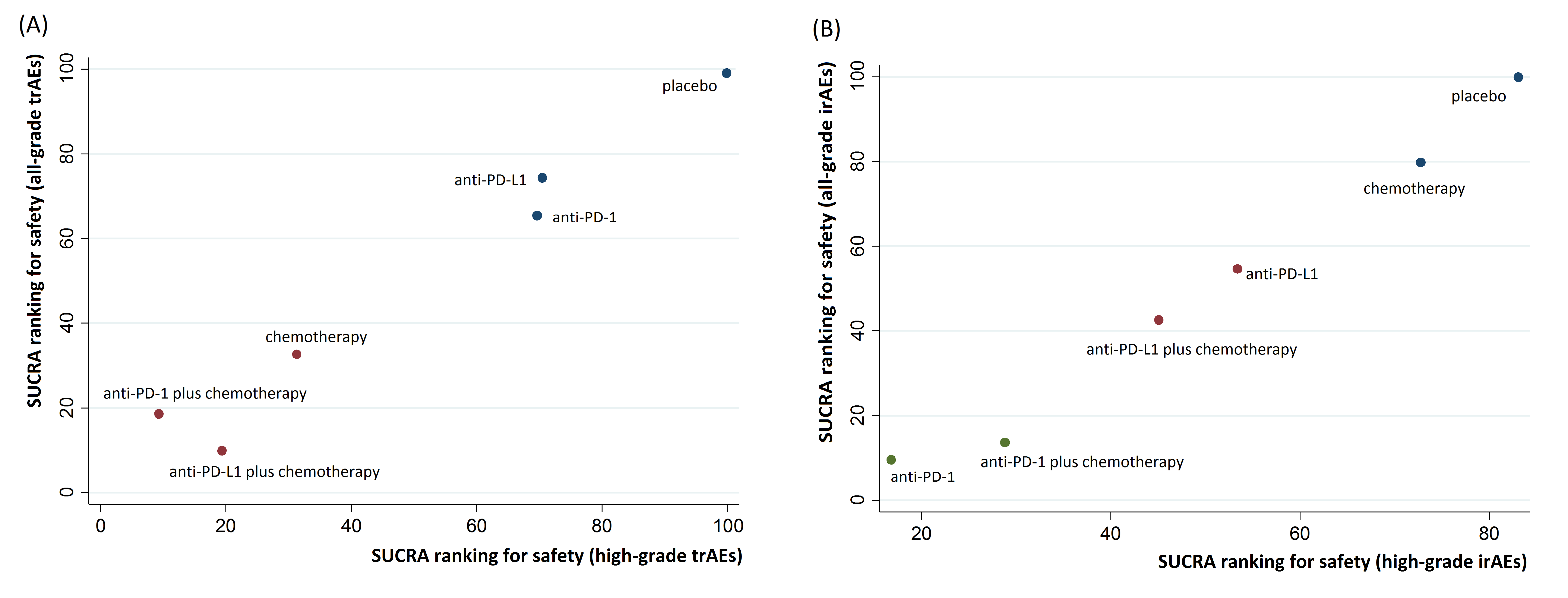

Supplement: Supplementary Figure 3 — Cluster SUCRA ranking plots (sensitivity analysis). (A) Cluster SUCRA ranking plot for safety on all-grade and high-grade trAEs (x-axis: SUCRA ranking for safety on high-grade trAEs; y-axis: SUCRA ranking for safety on all-grade trAEs). (B) Cluster SUCRA ranking plot for safety on all-grade and high-grade irAEs (x-axis: SUCRA ranking for safety on high-grade irAEs; y-axis: SUCRA ranking for safety on all-grade irAEs). For trAEs and irAEs, higher SUCRA ranking = safer treatment with lower risk of adverse events. irAEs, immune-related adverse events; trAEs, treatment-related adverse events. [file Image_3.TIF]
