# Supplementary material for: Comparative Safety of PD-1/PD-L1 Inhibitors for Cancer Patients: Systematic Review and Network Meta-Analysis
Source: Front Oncol. 2019 Oct 1;9:972. doi: 10.3389/fonc.2019.00972 (PMC6779807; doi:10.3389/fonc.2019.00972)
Supplement: Supplementary Table 1 — Search strategies. [file Table_1.DOCX]

**Supplementary Table 1. Search strategies.**

**PubMed**

| **#** | **Term** |
| --- | --- |
| 1 | Neoplasia [Title/Abstract] OR Tumor [Title/Abstract] OR Malignancy [Title/Abstract] OR Cancer* [Title/Abstract] OR Melanoma [Title/Abstract] |
| 2 | nivolumab[Supplementary Concept] OR nivolumab[Title/Abstract] OR MDX-1106[Title/Abstract] OR MDX1106[Title/Abstract] OR ONO-4538[Title/Abstract] OR ONO4538[Title/Abstract] OR BMS-936558[Title/Abstract] OR BMS936558[Title/Abstract] OR Opdivo[Title/Abstract] OR NIVO[Title/Abstract]  OR  pembrolizumab[Supplementary Concept] OR pembrolizumab [Title/Abstract] OR lambrolizumab [Supplementary Concept] OR lambrolizumab[Title/Abstract] OR keytruda[Title/Abstract] OR MK-3475[Title/Abstract] OR MK3475[Title/Abstract] OR SCH-900475[Title/Abstract] OR SCH900475[Title/Abstract]  OR  avelumab[Supplementary Concept] OR avelumab[Title/Abstract] OR bavenci[Title/Abstract] OR MSB-0010718C[Title/Abstract] OR MSB0010718C[Title/Abstract] OR MPDL-3280A [Title/Abstract] OR MPDL3280A [Title/Abstract]  OR  atezolizumab [Supplementary Concept] OR atezolizumab [Title/Abstract] OR Tecentriq[Title/Abstract] OR RG7446[Title/Abstract]) OR MPDL-3280A[Title/Abstract] OR MPDL3280A[Title/Abstract]  OR  durvalumab [Supplementary Concept] OR durvalumab[Title/Abstract] OR MEDI4736[Title/Abstract] OR MEDI-4736[Title/Abstract]  OR  PD-L1 [Title/Abstract] OR PDL1 [Title/Abstract] OR programmed cell death 1 ligand 1 [Title/Abstract] OR CD274 antigen [Title/Abstract] OR B7-H1 protein [Title/Abstract] OR B7H1 protein [Title/Abstract] OR B7-H1 protein [Title/Abstract] OR PD-1 [Title/Abstract] OR PD 1 [Title/Abstract] OR Programmed Cell Death 1 Receptor [Title/Abstract] OR CD279 [Title/Abstract] |
| 3 | random* [Title/Abstract] OR contro* [Title/Abstract] OR placebo [Title/Abstract] OR phase III [Title/Abstract] OR phase II [Title/Abstract] OR phase II/III [Title/Abstract] |
| 4 | #1 AND #2 AND #3 |

**EMBASE**

| **#** | **Term** |
| --- | --- |
| 1 | 'neoplasm'/exp |
| 2 | cancer OR tumor OR malignancy OR melanoma:ab |
| 3 | #1 OR #2 |
| 4 | 'nivolumab'/exp OR nivolumab OR 'mdx1106'/exp OR mdx1106 OR 'mdx-1106' OR 'ono4538'/exp OR ono4538 OR OR 'ono-4538' OR 'bms936558'/exp OR bms936558 OR 'bms-936558' OR 'opdivo'/exp OR opdivo OR 'NIVO'/exp OR NIVO  OR  'pembrolizumab'/exp OR pembrolizumab OR 'lambrolizumab'/exp OR lambrolizumab OR 'keytruda'/exp OR keytruda OR 'mk3475'/exp OR mk3475 OR 'mk-3475' OR 'sc900475'/exp OR sch900475 OR 'sch-900475'  OR  'avelumab'/exp OR avelumab OR 'bavenci'/exp OR bavenci OR 'msb0010718c'/exp OR msb0010718c OR 'msb-0010718c' OR 'mpdl3280a'/exp OR mpdl3280a OR 'mpdl-3280a'  OR  'atezolizumab'/exp OR atezolizumab OR 'mpdl3280a'/exp OR mpdl3280a OR 'mpdl-3280a' OR 'rg7446'/exp OR rg7446 OR 'rg-7446' OR 'tecntriq'/exp OR tecentriq  OR  'durvalumab'/exp OR durvalumab OR 'medi4736'/exp OR medi4736 OR 'medi-4736'  OR  'pd-l1' OR pdl1 OR 'programmed cell death 1 ligand 1' OR 'cd274 antigen' OR 'b7h1 protein' OR 'b7-h1 protein' OR 'pd-1' OR 'pd 1' OR 'programmed cell death 1 receptor' OR cd279  :ab |
| 5 | 'randomized controlled trial'/exp OR 'randomized controlled trial':ti,ab,kw OR 'randomized controlled trials'/exp OR 'randomized controlled trials':ti,ab,kw |
| 6 | 'editorial'/de OR 'letter'/de OR 'case report'/de |
| 7 | [animals]/lim NOT [humans]/lim |
| 8 | #6 OR #7 |
| 9 | #5 NOT #8 |
| 10 | #3 AND #4 AND #9 |
