# Supplementary material for: Comparative Safety of PD-1/PD-L1 Inhibitors for Cancer Patients: Systematic Review and Network Meta-Analysis
Source: Front Oncol. 2019 Oct 1;9:972. doi: 10.3389/fonc.2019.00972 (PMC6779807; doi:10.3389/fonc.2019.00972)
Supplement: Supplementary Table 2 — Risk of bias summary. [file Table_2.DOCX]

**Supplementary Table 2.** Risk of bias summary

| **Trial name** | **Random sequence generation** | **Allocation concealment** | **Blinding of participants and personnel** | **Blinding of outcome assessment** | **Incomplete outcome data** | **Selective reporting** | **Other sources of bias** |
| --- | --- | --- | --- | --- | --- | --- | --- |
| CheckMate 017 | Low | Low | High | Low | Low | High | Low |
| CheckMate 026 | Low | Low | High | Low | Low | High | Low |
| CheckMate 037 | Low | Low | High | Low | Low | High | Low |
| CheckMate 057 | Low | Low | High | Low | Low | High | Low |
| CheckMate 066 | Low | Low | Low | Low | Low | High | Low |
| CheckMate 227 | Low | Low | High | Low | Low | High | Low |
| IMpassion130 | Low | Low | Low | Low | Low | High | Low |
| IMpower133 | Low | Low | Low | Low | Low | High | Low |
| IMvigor211 | Low | Low | High | Low | Low | High | Low |
| KEYNOTE-002 | Low | Low | High | Low | Low | High | Low |
| KEYNOTE-010 | Low | Low | High | Low | Low | High | Low |
| KEYNOTE-021 | Low | Low | High | Low | Low | High | Low |
| KEYNOTE-024 | Low | Low | High | Low | Low | High | Low |
| KEYNOTE-040 | Low | Low | High | Low | Low | High | Low |
| KEYNOTE-045 | Low | Low | High | Low | Low | High | Low |
| KEYNOTE-054 | Low | Low | Low | Low | Low | High | Low |
| KEYNOTE-061 | Low | Low | High | Low | Low | High | Low |
| KEYNOTE-189 | Low | Low | Low | Low | Low | High | Low |
| KEYNOTE-407 | Low | Low | Low | Low | Low | High | Low |
| OAK | Low | Low | High | Low | Low | High | Low |
| ONO-4538-12, ATTRACTION-2 | Low | Low | Low | Low | Low | High | Low |
| PACIFIC study | Low | Low | Low | Low | Low | High | Low |
| POPLAR Study | Low | Low | High | Low | Low | High | Low |
