# Supplementary material for: Comparative Safety of PD-1/PD-L1 Inhibitors for Cancer Patients: Systematic Review and Network Meta-Analysis
Source: Front Oncol. 2019 Oct 1;9:972. doi: 10.3389/fonc.2019.00972 (PMC6779807; doi:10.3389/fonc.2019.00972)
Supplement: Supplementary Table 3 — Results of traditional pairwise meta-analyses for different outcomes. [file Table_3.DOCX]

**Supplementary Table 3.** Results of traditional pairwise meta-analyses for different outcomes

| **Comparison** | **Outcomes** | **Number of trials** | **Number of patients** | **Odds ratio (95% confidence interval)** | **I square value** |
| --- | --- | --- | --- | --- | --- |
| Anti-PD-1 vs chemotherapy | trAE 1-5 | 13 | 6000 | 0.37 (0.28 to 0.50) | 80% |
|  | trAE 3-5 | 13 | 6000 | 0.27 (0.20 to 0.37) | 83% |
|  | irAE 1-5 | 3 | 1305 | 3.49 (1.83 to 6.68) | 70% |
|  | irAE 3-5 | 3 | 1305 | 2.79 (0.69 to 11.19) | 73% |
| Anti-PD-1 vs placebo | trAE 1-5 | 2 | 1502 | 1.87 (1.48 to 2.36) | 0% |
|  | trAE 3-5 | 2 | 1502 | 3.49 (1.64 to 7.46) | 63% |
|  | irAE 1-5 | 1 | 1011 | 6.05 (4.24 to 8.63) | NE |
|  | irAE 3-5 | 1 | 1011 | 12.66 (3.87 to 41.38) | NE |
| Anti-PD-1 plus chemotherapy vs chemotherapy | trAE 1-5 | 1 | 121 | 1.47 (0.39 to 5.51) | NE |
|  | trAE 3-5 | 1 | 121 | 1.84 (0.85 to 3.98) | NE |
|  | irAE 1-5 | 3 | 1286 | 2.89 (1.75 to 4.76) | 51% |
|  | irAE 3-5 | 3 | 1286 | 2.27 (1.61 to 4.58) | 0% |
| Anti-PD-L1 vs chemotherapy | trAE 1-5 | 3 | 2366 | 0.29 (0.23 to 0.35) | 0% |
|  | trAE 3-5 | 3 | 2366 | 0.26 (0.20 to 0.34) | 44% |
|  | irAE 1-5 | 1 | 902 | 1.53 (1.13 to 2.06) | NE |
|  | irAE 3-5 | 1 | 902 | 2.69 (1.43 to 5.04) | NE |
| Anti-PD-L1 vs placebo | trAE 1-5 | 1 | 709 | 1.84 (1.33 to 2.53) | NE |
|  | trAE 3-5 | 1 | 709 | 2.60 (1.40 to 4.83) | NE |
|  | irAE 1-5 | 1 | 709 | 3.61 (2.16 to 6.04) | NE |
|  | irAE 3-5 | 1 | 709 | 1.10 (0.49 to 2.45) | NE |
| Anti-PD-L1 plus chemotherapy vs chemotherapy | trAE 1-5 | 2 | 1284 | 1.74 (1.06 to 2.88) | 0% |
|  | trAE 3-5 | 2 | 1284 | 1.29 (0.86 to 1.93) | 65% |
|  | irAE 1-5 | 2 | 1284 | 1.92 (1.53 to 2.40) | 0% |
|  | irAE 3-5 | 1 | 890 | 1.79 (1.01 to 3.20) | NE |

Note: All data was calculated using DerSimonian-Laird random-effects model; irAEs: immune-related adverse events; NE: not estimable due to limited number of trials; trAEs: treatment-related adverse events.
