# Supplementary material for: Comparative Safety of PD-1/PD-L1 Inhibitors for Cancer Patients: Systematic Review and Network Meta-Analysis
Source: Front Oncol. 2019 Oct 1;9:972. doi: 10.3389/fonc.2019.00972 (PMC6779807; doi:10.3389/fonc.2019.00972)
Supplement: Supplementary Table 4 — Evaluation of the quality of evidence using GRADE framework for outcomes. [file Table_4.DOCX]

**Supplementary Table 4.** Evaluation of the quality of evidence using GRADE framework for outcomes

| **Outcomes** | **Comparison** | | | **Direct evidence** | | **Indirect evidence** | | **Network meta-analysis** | |
| --- | --- | --- | --- | --- | --- | --- | --- | --- | --- |
|  |  |  |  | Odds ratio (95% confidence interval) | Quality of evidence | Odds ratio (95% credible interval) | Quality of evidence | Odds ratio (95% credible interval) | Quality of evidence |
| **All-grade trAEs** | chemotherapy | vs | placebo | - | - | 5.52 (3.07 to 9.95) | High | 5.52 (3.07 to 9.95) | High |
|  | anti-PD-L1 plus chemotherapy | vs | placebo | - | - | 9.62 (3.60 to 25.81) | High | 9.62 (3.60 to 25.81) | High |
|  | anti-PD-L1 | vs | placebo | 1.84 (1.33 to 2.53) | Moderate§ | 1.42 (0.62 to 3.24) | High | 1.62 (0.85 to 3.06) | Moderate¶ |
|  | anti-PD-1 plus chemotherapy | vs | placebo | - | - | 8.43 (1.54 to 49.48) | High | 8.43 (1.54 to 49.48) | High |
|  | anti-PD-1 | vs | placebo | 1.87 (1.48 to 2.36) | High | 2.46 (0.90 to 6.75) | High | 2.04 (1.17 to 3.59) | High |
|  | anti-PD-L1 plus chemotherapy | vs | chemotherapy | 1.74 (1.06 to 2.88) | High | Not estimable | Not estimable | 1.74 (0.79 to 3.85) | High |
|  | anti-PD-L1 | vs | chemotherapy | 0.29 (0.23 to 0.35) | Moderate* | 0.36 (0.13 to 1.05) | High | 0.29 (0.18 to 0.48) | Moderate¶ |
|  | anti-PD-1 plus chemotherapy | vs | chemotherapy | 1.47 (0.39 to 5.51) | Low*,§ | Not estimable | Not estimable | 1.53 (0.31 to 8.11) | Low*,§ |
|  | anti-PD-1 | vs | chemotherapy | 0.37 (0.28 to 0.50) | Low*,§ | 0.29 (0.09 to 0.93) | High | 0.37 (0.29 to 0.48) | Low*,§ |
|  | anti-PD-L1 | vs | anti-PD-L1 plus chemotherapy | - | - | 0.17 (0.07 to 0.43) | High | 0.17 (0.07 to 0.43) | High |
|  | anti-PD-1 plus chemotherapy | vs | anti-PD-L1 plus chemotherapy | - | - | 0.88 (0.15 to 5.55) | High | 0.88 (0.15 to 5.55) | High |
|  | anti-PD-1 | vs | anti-PD-L1 plus chemotherapy | - | - | 0.21 (0.09 to 0.49) | High | 0.21 (0.09 to 0.49) | High |
|  | anti-PD-1 plus chemotherapy | vs | anti-PD-L1 | - | - | 5.20 (0.99 to 29.89) | High | 5.20 (0.99 to 29.89) | High |
|  | anti-PD-1 | vs | anti-PD-L1 | - | - | 1.26 (0.74 to 2.17) | High | 1.26 (0.74 to 2.17) | High |
|  | anti-PD-1 | vs | anti-PD-1 plus chemotherapy | - | - | 0.24 (0.04 to 1.23) | High | 0.24 (0.04 to 1.23) | High |
| **High-grade trAEs** | chemotherapy | vs | placebo | - | - | 12.28 (5.46 to 27.68) | High | 12.28 (5.46 to 27.68) | High |
|  | anti-PD-L1 plus chemotherapy | vs | placebo | - | - | 15.52 (4.90 to 49.65) | High | 15.52 (4.90 to 49.65) | High |
|  | anti-PD-L1 | vs | placebo | 2.60 (1.40 to 4.83) | Moderate§ | 3.09 (1.04 to 9.16) | High | 2.95 (1.23 to 7.07) | Moderate¶ |
|  | anti-PD-1 plus chemotherapy | vs | placebo | - | - | 22.82 (4.67 to 112.20) | Moderate** | 22.82 (4.67 to 112.20) | Moderate** |
|  | anti-PD-1 | vs | placebo | 3.49 (1.64 to 7.46) | Moderate§ | 2.90 (0.74 to 11.29) | High | 3.39 (1.55 to 7.44) | Moderate¶ |
|  | anti-PD-L1 plus chemotherapy | vs | chemotherapy | 1.29 (0.86 to 1.93) | Moderate§ | Not estimable | Not estimable | 1.27 (0.55 to 2.90) | Moderate¶ |
|  | anti-PD-L1 | vs | chemotherapy | 0.26 (0.20 to 0.34) | Moderate* | 0.21 (0.05 to 0.91) | High | 0.24 (0.13 to 0.45) | Moderate¶ |
|  | anti-PD-1 plus chemotherapy | vs | chemotherapy | 1.84 (0.85 to 3.98) | Low*,§ | Not estimable | Not estimable | 1.86 (0.48 to 7.29) | Low*,§ |
|  | anti-PD-1 | vs | chemotherapy | 0.27 (0.20 to 0.37) | Low*,§ | 0.33 (0.07 to 1.60) | High | 0.28 (0.20 to 0.38) | Low*,§ |
|  | anti-PD-L1 | vs | anti-PD-L1 plus chemotherapy | - | - | 0.19 (0.07 to 0.54) | High | 0.19 (0.07 to 0.54) | High |
|  | anti-PD-1 plus chemotherapy | vs | anti-PD-L1 plus chemotherapy | - | - | 1.47 (0.30 to 7.30) | High | 1.47 (0.30 to 7.30) | High |
|  | anti-PD-1 | vs | anti-PD-L1 plus chemotherapy | - | - | 0.22 (0.09 to 0.53) | High | 0.22 (0.09 to 0.53) | High |
|  | anti-PD-1 plus chemotherapy | vs | anti-PD-L1 | - | - | 7.74 (1.71 to 35.00) | High | 7.74 (1.71 to 35.00) | High |
|  | anti-PD-1 | vs | anti-PD-L1 | - | - | 1.15 (0.58 to 2.29) | High | 1.15 (0.58 to 2.29) | High |
|  | anti-PD-1 | vs | anti-PD-1 plus chemotherapy | - | - | 0.15 (0.04 to 0.60) | High | 0.15 (0.04 to 0.60) | High |
| **All-grade irAEs** | chemotherapy | vs | placebo | - | - | 2.00 (0.78 to 4.82) | High | 2.00 (0.78 to 4.82) | High |
|  | anti-PD-L1 plus chemotherapy | vs | placebo | - | - | 3.90 (1.22 to 12.12) | High | 3.90 (1.22 to 12.12) | High |
|  | anti-PD-L1 | vs | placebo | 3.61 (2.16 to 6.04) | Moderate§ | 2.77 (0.94 to 8.17) | High | 3.29 (1.40 to 7.82) | Moderate¶ |
|  | anti-PD-1 plus chemotherapy | vs | placebo | - | - | 5.93 (1.84 to 17.09) | High | 5.93 (1.84 to 17.09) | High |
|  | anti-PD-1 | vs | placebo | 6.05 (4.24 to 8.63) | Moderate§ | 7.89 (2.51 to 24.76) | High | 6.60 (2.94 to 15.67) | Moderate¶ |
|  | anti-PD-L1 plus chemotherapy | vs | chemotherapy | 1.92 (1.53 to 2.40) | High | Not estimable | Not estimable | 1.94 (0.98 to 3.95) | High |
|  | anti-PD-L1 | vs | chemotherapy | 1.53 (1.13 to 2.06) | Low*,§ | 2.00 (0.63 to 6.37) | High | 1.64 (0.74 to 3.89) | Low*,§ |
|  | anti-PD-1 plus chemotherapy | vs | chemotherapy | 2.89 (1.75 to 4.76) | Low*,§ | Not estimable | Not estimable | 2.95 (1.52 to 5.51) | Low*,§ |
|  | anti-PD-1 | vs | chemotherapy | 3.49 (1.83 to 6.68) | Low*,§ | 2.56 (0.75 to 8.79) | High | 3.30 (1.93 to 6.32) | Low*,§ |
|  | anti-PD-L1 | vs | anti-PD-L1 plus chemotherapy | - | - | 0.84 (0.29 to 2.55) | High | 0.84 (0.29 to 2.55) | High |
|  | anti-PD-1 plus chemotherapy | vs | anti-PD-L1 plus chemotherapy | - | - | 1.52 (0.57 to 3.78) | High | 1.52 (0.57 to 3.78) | High |
|  | anti-PD-1 | vs | anti-PD-L1 plus chemotherapy | - | - | 1.70 (0.72 to 4.47) | High | 1.70 (0.72 to 4.47) | High |
|  | anti-PD-1 plus chemotherapy | vs | anti-PD-L1 | - | - | 1.80 (0.60 to 4.84) | High | 1.80 (0.60 to 4.84) | High |
|  | anti-PD-1 | vs | anti-PD-L1 | - | - | 2.01 (0.84 to 5.09) | High | 2.01 (0.84 to 5.09) | High |
|  | anti-PD-1 | vs | anti-PD-1 plus chemotherapy | - | - | 1.12 (0.50 to 2.89) | High | 1.12 (0.50 to 2.89) | High |
| **High-grade irAEs** | chemotherapy | vs | placebo | - | - | 1.54 (0.14 to 17.26) | High | 1.54 (0.14 to 17.26) | High |
|  | anti-PD-L1 plus chemotherapy | vs | placebo | - | - | 2.81 (0.09 to 94.86) | Moderate** | 2.81 (0.09 to 94.86) | Moderate** |
|  | anti-PD-L1 | vs | placebo | 1.10 (0.49 to 2.45) | Moderate§ | 14.28 (1.25 to 163.27) | Moderate** | 2.25 (0.25 to 20.10) | Moderate¶ |
|  | anti-PD-1 plus chemotherapy | vs | placebo | - | - | 4.30 (0.24 to 79.60) | Moderate** | 4.30 (0.24 to 79.60) | Moderate** |
|  | anti-PD-1 | vs | placebo | 12.66 (3.87 to 41.38) | Moderate§ | 0.97 (0.10 to 9.47) | High | 6.36 (0.74 to 66.54) | Moderate¶ |
|  | anti-PD-L1 plus chemotherapy | vs | chemotherapy | 1.79 (1.01 to 3.20) | Moderate§ | Not estimable | Not estimable | 1.82 (0.14 to 22.92) | Moderate¶ |
|  | anti-PD-L1 | vs | chemotherapy | 2.69 (1.43 to 5.04) | Low*,§ | 0.21 (0.02 to 2.49) | High | 1.46 (0.16 to 12.67) | Low*,§ |
|  | anti-PD-1 plus chemotherapy | vs | chemotherapy | 2.27 (1.61 to 4.58) | Moderate* | Not estimable | Not estimable | 2.79 (0.56 to 14.36) | Moderate¶ |
|  | anti-PD-1 | vs | chemotherapy | 2.79 (0.69 to 11.19) | Low*,§ | 30.95 (2.27 to 421.05) | Moderate** | 4.13 (0.97 to 20.05) | Low*,§ |
|  | anti-PD-L1 | vs | anti-PD-L1 plus chemotherapy | - | - | 0.80 (0.03 to 22.19) | High | 0.80 (0.03 to 22.19) | High |
|  | anti-PD-1 plus chemotherapy | vs | anti-PD-L1 plus chemotherapy | - | - | 1.53 (0.07 to 31.33) | High | 1.53 (0.07 to 31.33) | High |
|  | anti-PD-1 | vs | anti-PD-L1 plus chemotherapy | - | - | 2.27 (0.13 to 46.77) | High | 2.27 (0.13 to 46.77) | High |
|  | anti-PD-1 plus chemotherapy | vs | anti-PD-L1 | - | - | 1.91 (0.13 to 30.00) | High | 1.91 (0.13 to 30.00) | High |
|  | anti-PD-1 | vs | anti-PD-L1 | - | - | 2.84 (0.29 to 33.13) | High | 2.84 (0.29 to 33.13) | High |
|  | anti-PD-1 | vs | anti-PD-1 plus chemotherapy | - | - | 1.49 (0.17 to 14.59) | High | 1.49 (0.17 to 14.59) | High |

* Inadequate concealment of allocation or unblinded unvalidated assessment by the physician; † Heterogeneity of outcome definition or outcome assessing between each trial;

‡Wide confidence intervals and few events; § Large I^2^; ¶ Contributing direct evidence of moderate quality; ** Imprecision.
