# Supplementary material for: Comparative Safety of PD-1/PD-L1 Inhibitors for Cancer Patients: Systematic Review and Network Meta-Analysis
Source: Front Oncol. 2019 Oct 1;9:972. doi: 10.3389/fonc.2019.00972 (PMC6779807; doi:10.3389/fonc.2019.00972)
Supplement: Supplementary Table 5 — Median ranks on safety (rank 1–6 on each scale) and 95% CrIs. [file Table_5.docx]

**Supplementary Table 5.** Median ranks on safety (rank 1-6 on each scale) and 95% CrIs.

| **Types of analysis** | **Treatment** | **Placebo** | **Chemotherapy** | **Anti-PD-L1 plus chemotherapy** | **Anti-PD-L1** | **Anti-PD-1 plus chemotherapy** | **Anti-PD-1** |
| --- | --- | --- | --- | --- | --- | --- | --- |
| Main analysis | All-grade trAEs rank (95% CrIs) | 1 (1 to 2) | 4 (4 to 5) | 6 (4 to 6) | 2 (1 to 3) | 5 (3 to 6) | 3 (2 to 4) |
|  | High-grade trAEs rank (95% CrIs) | 1 (1 to 1) | 4 (4 to 6) | 5 (4 to 6) | 2 (2 to 3) | 6 (4 to 6) | 3 (2 to 3) |
|  | All-grade irAEs rank (95% CrIs) | 1 (1 to 2) | 2 (1 to 3) | 4 (2 to 6) | 3 (2 to 5) | 5 (3 to 6) | 6 (4 to 6) |
|  | High-grade irAEs rank (95% CrIs) | 1 (1 to 5) | 2 (1 to 4) | 4 (1 to 6) | 3 (1 to 6) | 5 (1 to 6) | 5 (3 to 6) |
| Sensitivity analysis | All-grade trAEs rank (95% CrIs) | 1 (1 to 2) | 4 (4 to 5) | 6 (4 to 6) | 2 (1 to 3) | 5 (3 to 6) | 3 (2 to 4) |
|  | High-grade trAEs rank (95% CrIs) | 1 (1 to 1) | 4 (4 to 6) | 5 (4 to 6) | 2 (2 to 3) | 6 (4 to 6) | 3 (2 to 3) |
|  | All-grade irAEs rank (95% CrIs) | 1 (1 to 2) | 2 (1 to 3) | 4 (2 to 6) | 3 (2 to 5) | 5 (3 to 6) | 6 (4 to 6) |
|  | High-grade irAEs rank (95% CrIs) | 1 (1 to 5) | 2 (1 to 4) | 4 (1 to 6) | 3 (1 to 6) | 5 (1 to 6) | 5 (3 to 6) |

CrIs: credible intervals; irAEs: immune-related adverse events; trAEs: treatment-related adverse events.
