# Supplementary material for: Comparative Safety of PD-1/PD-L1 Inhibitors for Cancer Patients: Systematic Review and Network Meta-Analysis
Source: Front Oncol. 2019 Oct 1;9:972. doi: 10.3389/fonc.2019.00972 (PMC6779807; doi:10.3389/fonc.2019.00972)
Supplement: Supplementary Table 6 — Nodesplit analysis of network meta-analysis for treatment-related adverse events and immune-related adverse events. [file Table_6.DOCX]

**Supplementary Table 6.** Nodesplit analysis of network meta-analysis for treatment-related adverse events and immune-related adverse events

| **Nodes** | **Direct, OR (95% CI)** | **Indirect, OR (95% CI)** | **Difference, OR (95% CI)** | **P**** |
| --- | --- | --- | --- | --- |
| **All-grade trAEs** |  |  |  |  |
| anti-PD-L1, placebo | 1.84 (0.79 to 4.24) | 1.42 (0.62 to 3.24) | 1.29 (0.40 to 4.18) | 0.67 |
| anti-PD-1, placebo | 1.91 (1.05 to 3.48) | 2.46 (0.90 to 6.75) | 0.78 (0.24 to 2.51) | 0.67 |
| anti-PD-L1 plus chemotherapy, chemotherapy* | 1.72 (0.83 to 3.58) | 0.04 (NE) | 48.19 (NE) | 0.98 |
| anti-PD-L1, chemotherapy | 0.28 (0.17 to 0.47) | 0.36 (0.13 to 1.05) | 0.77 (0.24 to 2.51) | 0.67 |
| anti-PD-1 plus chemotherapy, chemotherapy* | 1.47 (0.32 to 6.70) | 0.04 (NE) | 41.94 (NE) | 0.99 |
| anti-PD-1, chemotherapy | 0.38 (0.30 to 0.48) | 0.29 (0.09 to 0.93) | 1.29 (0.40 to 4.17) | 0.67 |
| **High-grade trAEs** |  |  |  |  |
| anti-PD-L1, placebo | 2.60 (0.80 to 8.41) | 3.09 (1.04 to 9.16) | 0.84 (0.17 to 4.16) | 0.83 |
| anti-PD-1, placebo | 3.47 (1.50 to 8.03) | 2.90 (0.74 to 11.29) | 1.20 (0.24 to 5.91) | 0.83 |
| anti-PD-L1 plus chemotherapy, chemotherapy* | 1.26 (0.61 to 2.61) | 0.01 (NE) | 176.80 (NE) | 0.96 |
| anti-PD-L1, chemotherapy | 0.25 (0.13 to 0.46) | 0.21 (0.05 to 0.91) | 1.19 (0.24 to 5.89) | 0.83 |
| anti-PD-1 plus chemotherapy, chemotherapy* | 1.84 (0.53 to 6.31) | 0.01 (NE) | 258.86 (NE) | 0.97 |
| anti-PD-1, chemotherapy | 0.28 (0.21 to 0.37) | 0.33 (0.07 to 1.60) | 0.84 (0.17 to 4.15) | 0.83 |
| **All-grade irAEs** |  |  |  |  |
| anti-PD-L1, placebo | 3.61 (1.65 to 7.90) | 2.77 (0.94 to 8.17) | 1.31 (0.34 to 4.96) | 0.70 |
| anti-PD-1, placebo | 6.05 (3.04 to 12.04) | 7.89 (2.51 to 24.76) | 0.77 (0.20 to 2.91) | 0.70 |
| anti-PD-L1 plus chemotherapy, chemotherapy* | 1.94 (1.34 to 2.79) | 0.23 (NE) | 8.45 (NE) | 0.98 |
| anti-PD-L1, chemotherapy | 1.53 (0.79 to 2.96) | 2.00 (0.63 to 6.37) | 0.77 (0.20 to 2.91) | 0.70 |
| anti-PD-1 plus chemotherapy, chemotherapy* | 2.92 (1.94 to 4.40) | 0.18 (NE) | 16.26 (NE) | 0.98 |
| anti-PD-1, chemotherapy | 3.34 (2.01 to 5.56) | 2.56 (0.75 to 8.79) | 1.30 (0.34 to 4.96) | 0.70 |
| **High-grade irAEs** |  |  |  |  |
| anti-PD-L1, placebo | 1.10 (0.26 to 4.68) | 14.28 (1.25 to 163.27) | 0.08 (0.00 to 1.31) | 0.08 |
| anti-PD-1, placebo | 12.66 (2.33 to 68.69) | 0.97 (0.10 to 9.47) | 13.00 (0.76 to 221.43) | 0.08 |
| anti-PD-L1 plus chemotherapy, chemotherapy* | 1.79 (0.28 to 11.48) | 0.39 (NE) | 4.65 (NE) | 1.00 |
| anti-PD-L1, chemotherapy | 2.69 (0.69 to 10.48) | 0.21 (0.02 to 2.49) | 13.00 (0.76 to 221.40) | 0.08 |
| anti-PD-1 plus chemotherapy, chemotherapy* | 2.64 (0.77 to 9.10) | 0.31 (NE) | 8.61 (NE) | 0.99 |
| anti-PD-1, chemotherapy | 2.38 (0.79 to 7.19) | 30.95 (2.27 to 421.05) | 0.08 (0.00 to 1.31) | 0.08 |

* All the evidence about these contrasts comes from the trials which directly compare them.

** P equal or less than 0.05 indicates a significant inconsistency between the direct effect and indirect effects.

NE: not estimable.
