# Supplementary material for: Comparative Safety of PD-1/PD-L1 Inhibitors for Cancer Patients: Systematic Review and Network Meta-Analysis
Source: Front Oncol. 2019 Oct 1;9:972. doi: 10.3389/fonc.2019.00972 (PMC6779807; doi:10.3389/fonc.2019.00972)
Supplement: Supplementary Table 7 — Evaluation of inconsistency using loop-specific heterogeneity estimates. [file Table_7.DOCX]

**Supplementary Table 7.** Evaluation of inconsistency using loop-specific heterogeneity estimates

| **Loop** | **ROR (95% CI)** | **P value** |
| --- | --- | --- |
| **All-grade trAEs** |  |  |
| Anti-PD-1, anti-PD-L1, chemotherapy, placebo | 1.29 (1.00 to 4.34) | 0.68 |
| **High-grade trAEs** |  |  |
| Anti-PD-1, anti-PD-L1, chemotherapy, placebo | 1.20 (1.00 to 6.65) | 0.84 |
| **All-grade irAEs** |  |  |
| Anti-PD-1, anti-PD-L1, chemotherapy, placebo | 1.37 (1.00 to 11.56) | 0.78 |
| **High-grade irAEs** |  |  |
| Anti-PD-1, anti-PD-L1, chemotherapy, placebo | 11.11 (1.00 to 931.28) | 0.29 |
