# Supplementary material for: Comparative Safety of PD-1/PD-L1 Inhibitors for Cancer Patients: Systematic Review and Network Meta-Analysis
Source: Front Oncol. 2019 Oct 1;9:972. doi: 10.3389/fonc.2019.00972 (PMC6779807; doi:10.3389/fonc.2019.00972)
Supplement: Supplementary Table 8 — Meta-regression for treatment-related adverse events and immune-related adverse events. [file Table_8.DOCX]

**Supplementary Table 8 Meta-regression for treatment-related adverse events and immune-related adverse events**

| Variable | Regression coefficient, Mean (95% CrI) | |
| --- | --- | --- |
|  | All-grade | High-grade |
| **Treatment-related adverse events** |  |  |
| Median age | 0.03 (-0.11 to 0.16) | -0.07 (-0.25 to 0.11) |
| Percentage of male | 2.44 (-11.25 to 16.05) | -7.58 (-26.03 to 10.82) |
| Line of treatment | -19.27 (-80.20 to 30.92) | -6.09 (-72.11 to 67.67) |
| Tumor histology | 0.25 (-1.08 to 1.58) | -0.18 (-1.99 to 1.63) |
| Whether double-blind was used | 0.52 (-195.80 to 197.00) | 0.24 (-195.50 to 194.90) |
| **Immune-related adverse events** |  |  |
| Median age | 0.03 (-0.20 to 0.28) | -0.25 (-0.70 to 0.23) |
| Percentage of male | 3.78 (-22.78 to 31.79) | -28.48 (-75.10 to 25.69) |
| Line of treatment | -8.15 (-60.55 to 67.39) | -3.71 (-81.62 to 84.78) |
| Tumor histology | 0.32 (-2.00 to 2.72) | -2.53 (-6.87 to 2.10) |
| Whether double-blind was used | -0.01 (-196.80 to 195.80) | -0.15 (-195.60 to 195.50) |
