# Supplementary material for: Comparative Safety of PD-1/PD-L1 Inhibitors for Cancer Patients: Systematic Review and Network Meta-Analysis
Source: Front Oncol. 2019 Oct 1;9:972. doi: 10.3389/fonc.2019.00972 (PMC6779807; doi:10.3389/fonc.2019.00972)
Supplement: Supplementary Table 9 — Subgroup analysis: median ranks on safety (rank 1–6 on each scale) and 95% CrIs. [file Table_9.DOCX]

**Supplementary Table 9.** Subgroup analysis: **m**edian ranks on safety (rank 1-6 on each scale) and 95% CrIs.

| **Types of subgroups** | **Treatment** | **Placebo** | **Chemotherapy** | **Anti-PD-L1 plus chemotherapy** | **Anti-PD-L1** | **Anti-PD-1 plus chemotherapy** | **Anti-PD-1** |
| --- | --- | --- | --- | --- | --- | --- | --- |
| NSCLC | All-grade trAEs rank (95% CrIs) | 1 (1 to 2) | 4 (4 to 5) | NA | 2 (2 to 3) | 5 (3 to 5) | 3 (1 to 3) |
|  | High-grade trAEs rank (95% CrIs) | 1 (1 to 3) | 4 (4 to 5) | NA | 2 (1 to 3) | 5 (3 to 5) | 2 (1 to 3) |
|  | All-grade irAEs rank (95% CrIs) | NA | 1 (1 to 2) | NA | NA | 2 (1 to 3) | 3 (2 to 3) |
|  | High-grade irAEs rank (95% CrIs) | NA | 1 (1 to 2) | NA | NA | 2 (1 to 3) | 3 (2 to 3) |
| Melanoma | All-grade trAEs rank (95% CrIs) | 1 (1 to 3) | 3 (2 to 3) | NA | NA | NA | 2 (1 to 3) |
|  | High-grade trAEs rank (95% CrIs) | 1 (1 to 1) | 3 (2 to 3) | NA | NA | NA | 2 (2 to 3) |
|  | All-grade irAEs rank (95% CrIs) | NA | NA | NA | NA | NA | NA |
|  | High-grade irAEs rank (95% CrIs) | NA | NA | NA | NA | NA | NA |
| First-line | All-grade trAEs rank (95% CrIs) | NA | 3 (3 to 5) | 5 (3 to 5) | 1 (1 to 4) | 4 (1 to 5) | 2 (1 to 3) |
|  | High-grade trAEs rank (95% CrIs) | NA | 3 (3 to 5) | 4 (3 to 5) | 1 (1 to 3) | 5 (3 to 5) | 2 (1 to 2) |
|  | All-grade irAEs rank (95% CrIs) | NA | 1 (1 to 3) | 3 (1 to 5) | 2 (1 to 5) | 4 (2 to 5) | 5 (2 to 5) |
|  | High-grade irAEs rank (95% CrIs) | NA | 2 (1 to 3) | 3 (1 to 5) | 4 (1 to 5) | 4 (1 to 5) | 4 (1 to 5) |
| Second-line or higher | All-grade trAEs rank (95% CrIs) | 1 (1 to 2) | 4 (4 to 4) | NA | 2 (1 to 3) | NA | 3 (2 to 3) |
|  | High-grade trAEs rank (95% CrIs) | 1 (1 to 2) | 4 (4 to 4) | NA | 2 (1 to 3) | NA | 3 (2 to 3) |
|  | All-grade irAEs rank (95% CrIs) | NA | NA | NA | NA | NA | NA |
|  | High-grade irAEs rank (95% CrIs) | NA | NA | NA | NA | NA | NA |

CrIs: credible intervals; irAEs: immune-related adverse events; NA: not available; NSCLC: non-small cell lung cancer; trAEs: treatment-related adverse events.
