# Supplementary material for: Comparative Safety of PD-1/PD-L1 Inhibitors for Cancer Patients: Systematic Review and Network Meta-Analysis
Source: Front Oncol. 2019 Oct 1;9:972. doi: 10.3389/fonc.2019.00972 (PMC6779807; doi:10.3389/fonc.2019.00972)
Supplement: Supplementary Table 10 — SUCRA rankings in the subgroup analysis based on type of cancer and line of treatment. [file Table_10.DOCX]

**Supplementary Table 10.** SUCRA rankings in the subgroup analysis based on type of cancer and line of treatment.

|  | **Type of treatment (SUCRA value)** | | | | |
| --- | --- | --- | --- | --- | --- |
|  | **Rank 1** | **Rank 2** | **Rank 3** | **Rank 4** | **Rank 5** |
| NSCLC |  |  |  |  |  |
| trAE1-5 | Placebo (99.6) | Anti-PD-L1 (69.7) | Anti-PD-1 (54.8) | Chemotherapy (17.7) | Anti-PD-1 plus chemotherapy (8.1) |
| trAE3-5 | Placebo (94.9) | Anti-PD-1 (66.3) | Anti-PD-L1 (63.6) | Chemotherapy (20.0) | Anti-PD-1 plus chemotherapy (5.3) |
| irAE1-5 | Chemotherapy (100.0) | Anti-PD-1 plus chemotherapy (48.3) | Anti-PD-1 (1.7) |  |  |
| irAE3-5 | Chemotherapy (99.9) | Anti-PD-1 plus chemotherapy (47.5) | Anti-PD-1 (2.6) |  |  |
| Melanoma |  |  |  |  |  |
| trAE1-5 | Placebo (100.0) | Anti-PD-1 (49.1) | Chemotherapy (0.9) |  |  |
| trAE3-5 | Placebo (100.0) | Anti-PD-1 (50.0) | Chemotherapy (0) |  |  |
| irAE1-5 | NE |  |  |  |  |
| irAE3-5 | NE |  |  |  |  |
| First-line |  |  |  |  |  |
| trAE1-5 | Anti-PD-L1 (90.0) | Anti-PD-1 (82.2) | Chemotherapy (39.7) | Anti-PD-1 plus chemotherapy (24.5) | Anti-PD-L1 plus chemotherapy (13.7) |
| trAE3-5 | Anti-PD-L1 (88.9) | Anti-PD-1 (85.9) | Chemotherapy (41.6) | Anti-PD-L1 plus chemotherapy (23.5) | Anti-PD-1 plus chemotherapy (10.1) |
| irAE1-5 | Chemotherapy (96.2) | Anti-PD-L1 (67.9) | Anti-PD-L1 plus chemotherapy (51.8) | Anti-PD-1 plus chemotherapy (25.9) | Anti-PD-1 (8.2) |
| irAE3-5 | Chemotherapy (85.4) | Anti-PD-L1 plus chemotherapy (52.8) | Anti-PD-1 (40.1) | Anti-PD-L1 (37.1) | Anti-PD-1 plus chemotherapy (34.6) |
| Second-line or higher |  |  |  |  |  |
| trAE1-5 | Placebo (98.3) | Anti-PD-L1 (61.9) | Anti-PD-1 (39.7) | Chemotherapy (0) |  |
| trAE3-5 | Placebo (99.5) | Anti-PD-L1 (55.1) | Anti-PD-1 (45.4) | Chemotherapy (0) |  |
| irAE1-5 | NE |  |  |  |  |
| irAE3-5 | NE |  |  |  |  |

irAEs: immune-related adverse events; NE: not estimable due to limited number of trials; NSCLC: non-small cell lung cancer; SUCRA: surface under the cumulative ranking; trAEs: treatment-related adverse events.
