# Supplementary material for: Comparative Safety of PD-1/PD-L1 Inhibitors for Cancer Patients: Systematic Review and Network Meta-Analysis
Source: Front Oncol. 2019 Oct 1;9:972. doi: 10.3389/fonc.2019.00972 (PMC6779807; doi:10.3389/fonc.2019.00972)
Supplement: Supplementary Table 11 — Pooled incidence along with corresponding 95% credible intervals of treatment based on type of cancer and line of treatment. [file Table_11.DOCX]

**Supplementary table 11.** Pooled incidence along with corresponding 95% credible intervals of treatment based on type of cancer and line of treatment

| **Subgroup** | **Treatment** | **trAE 1-5** | **trAE 3-5** | **irAE 1-5** | **irAE 3-5** |
| --- | --- | --- | --- | --- | --- |
| NSCLC | Chemotherapy | 92.5% (79.2% to 100.1%) | 10.3% (6.8% to 11.0%) | NE | NE |
|  | Anti-PD-L1 | 69.2% (52.3% to 83.3%) | 8.1% (3.6% to 10.4%) | NE | NE |
|  | Anti-PD-1 plus chemotherapy | 97.0% (69.6% to 105.0%) | 10.7% (6.7% to 11.1%) | 12.1% (7.9% to 14.5%)* | 5.7% (3.4% to 7.0%)* |
|  | Anti-PD-1 | 73.3% (50.5% to 89.3%) | 8.0% (2.5% to 10.6%) | 14.6% (9.1% to 16.0%)* | 7.4% (5.0% to 7.7%)* |
| Melanoma | Chemotherapy | 93.4% (57.3% to 117.1%) | 6.2% (4.3% to 6.7%) | NE | NE |
|  | Anti-PD-1 | 85.0% (52.0% to 110.2%) | 5.6% (3.5% to 6.5%) | NE | NE |
| First-line | Anti-PD-L1 plus chemotherapy | 111.0% (62.7% to 147.5%)* | 43.8% (28.2% to 57.5%)* | 22.6% (13.9% to 29.1%)* | 3.9% (0.7% to 5.8%)* |
|  | Anti-PD-L1 | 37.8% (10.3% to 95.6%)* | 19.0% (7.5% to 38.7%)* | 20.7% (8.9% to 29.8%)* | 4.4% (1.0% to 5.9%)* |
|  | Anti-PD-1 plus chemotherapy | 105.8% (31.2% to 160.9%)* | 50.7% (26.1% to 68.2%)* | 25.5% (18.3% to 30.0%)* | 4.4% (2.0% to 5.6%)* |
|  | Anti-PD-1 | 45.5% (27.9% to 68.5%)* | 19.9% (13.9% to 27.8%)* | 27.6% (20.6% to 31.9%)* | 4.5% (1.9% to 5.8%)* |
| Second-line or higher | Chemotherapy | 82.3% (72.1% to 88.9%) | 8.0% (7.2% to 8.4%) | NE | NE |
|  | Anti-PD-L1 | 60.5% (43.5% to 74.7%) | 6.4% (4.2% to 7.8%) | NE | NE |
|  | Anti-PD-1 | 65.6% (52.1% to 77.0%) | 6.6% (4.8% to 7.7%) | NE | NE |

Note: The assumed placebo risk was used to estimate the incidence. When the placebo risk is not available, chemotherapy risk(*) was used.

irAEs: immune-related adverse events; NE: not estimable due to limited number of trials; NSCLC: non-small cell lung cancer; trAEs: treatment-related adverse events.
