# Supplementary material for: Comparative Safety of PD-1/PD-L1 Inhibitors for Cancer Patients: Systematic Review and Network Meta-Analysis
Source: Front Oncol. 2019 Oct 1;9:972. doi: 10.3389/fonc.2019.00972 (PMC6779807; doi:10.3389/fonc.2019.00972)
Supplement: Supplementary Table 12 — Bayesian network meta-analysis based on type of cancer and line of treatment. [file Table_12.docx]

**Supplementary Table 12.** Bayesian network meta-analysis based on type of cancer and line of treatment

| **Subgroup** | **Outcomes** | **Comparison** | | | **Odds ratio (95% credible interval)** |
| --- | --- | --- | --- | --- | --- |
| NSCLC | trAE 1-5 | Chemotherapy | vs | Placebo | **6.42 (2.87 to 14.82)** |
|  |  | Anti-PD-L1 | vs | Placebo | 1.84 (0.96 to 3.54) |
|  |  | Anti-PD-1 plus chemotherapy | vs | Placebo | **9.82 (1.87 to 56.71)** |
|  |  | Anti-PD-1 | vs | Placebo | 2.19 (0.90 to 5.07) |
|  |  | Anti-PD-L1 | vs | Chemotherapy | **0.29 (0.17 to 0.47)** |
|  |  | Anti-PD-1 plus chemotherapy | vs | Chemotherapy | 1.52 (0.36 to 7.23) |
|  |  | Anti-PD-1 | vs | Chemotherapy | **0.34 (0.25 to 0.43)** |
|  |  | Anti-PD-1 plus chemotherapy | vs | Anti-PD-L1 | **5.34 (1.15 to 27.27)** |
|  |  | Anti-PD-1 | vs | Anti-PD-L1 | 1.19 (0.66 to 2.04) |
|  |  | Anti-PD-1 | vs | Anti-PD-1 plus chemotherapy | **0.22 (0.05 to 0.97)** |
|  | trAE 3-5 | Chemotherapy | vs | Placebo | **12.57 (1.57 to 101.50)** |
|  |  | Anti-PD-L1 | vs | Placebo | 2.67 (0.48 to 14.89) |
|  |  | Anti-PD-1 plus chemotherapy | vs | Placebo | **23.35 (1.53 to 367.40)** |
|  |  | Anti-PD-1 | vs | Placebo | 2.61 (0.29 to 22.66) |
|  |  | Anti-PD-L1 | vs | Chemotherapy | **0.21 (0.06 to 0.69)** |
|  |  | Anti-PD-1 plus chemotherapy | vs | Chemotherapy | 1.86 (0.32 to 11.10) |
|  |  | Anti-PD-1 | vs | Chemotherapy | **0.21 (0.11 to 0.38)** |
|  |  | Anti-PD-1 plus chemotherapy | vs | Anti-PD-L1 | **8.75 (1.05 to 74.65)** |
|  |  | Anti-PD-1 | vs | Anti-PD-L1 | 0.98 (0.25 to 3.73) |
|  |  | Anti-PD-1 | vs | Anti-PD-1 plus chemotherapy | **0.11 (0.02 to 0.72)** |
|  | irAE 1-5 | Anti-PD-1 plus chemotherapy | vs | Chemotherapy | 2.93 (0.95 to 8.53) |
|  |  | Anti-PD-1 | vs | Chemotherapy | **8.88 (1.27 to 63.50)** |
|  |  | Anti-PD-1 | vs | Anti-PD-1 plus chemotherapy | 3.06 (0.33 to 29.56) |
|  | irAE 3-5 | Anti-PD-1 plus chemotherapy | vs | Chemotherapy | 2.79 (0.80 to 10.00) |
|  |  | Anti-PD-1 | vs | Chemotherapy | **23.65 (1.89 to 1053.00)** |
|  |  | Anti-PD-1 | vs | Anti-PD-1 plus chemotherapy | 8.58 (0.50 to 442.80) |
| Melanoma | trAE 1-5 | Chemotherapy | vs | Placebo | 2.40 (0.77 to 7.72) |
|  |  | Anti-PD-1 | vs | Placebo | 1.80 (0.65 to 4.98) |
|  |  | Anti-PD-1 | vs | Chemotherapy | 0.75 (0.43 to 1.27) |
|  | trAE 3-5 | Chemotherapy | vs | Placebo | **10.07 (1.77 to 57.63)** |
|  |  | Anti-PD-1 | vs | Placebo | **5.03 (1.05 to 24.11)** |
|  |  | Anti-PD-1 | vs | Chemotherapy | 0.50 (0.23 to 1.08) |
| First-line | trAE 1-5 | Anti-PD-L1 plus chemotherapy | vs | Chemotherapy | 1.74 (0.56 to 5.36) |
|  |  | Anti-PD-L1 | vs | Chemotherapy | 0.28 (0.06 to 1.20) |
|  |  | Anti-PD-1 plus chemotherapy | vs | Chemotherapy | 1.53 (0.22 to 11.42) |
|  |  | Anti-PD-1 | vs | Chemotherapy | **0.35 (0.19 to 0.64)** |
|  |  | Anti-PD-L1 | vs | Anti-PD-L1 plus chemotherapy | 0.16 (0.02 to 1.02) |
|  |  | Anti-PD-1 plus chemotherapy | vs | Anti-PD-L1 plus chemotherapy | 0.88 (0.09 to 8.83) |
|  |  | Anti-PD-1 | vs | Anti-PD-L1 plus chemotherapy | **0.20 (0.06 to 0.73)** |
|  |  | Anti-PD-1 plus chemotherapy | vs | Anti-PD-L1 | 5.55 (0.48 to 66.39) |
|  |  | Anti-PD-1 | vs | Anti-PD-L1 | 1.28 (0.26 to 6.31) |
|  |  | Anti-PD-1 | vs | Anti-PD-1 plus chemotherapy | 0.23 (0.03 to 1.76) |
|  | trAE 3-5 | Anti-PD-L1 plus chemotherapy | vs | Chemotherapy | 1.28 (0.57 to 2.80) |
|  |  | Anti-PD-L1 | vs | Chemotherapy | **0.32 (0.11 to 0.98)** |
|  |  | Anti-PD-1 plus chemotherapy | vs | Chemotherapy | 1.86 (0.50 to 6.95) |
|  |  | Anti-PD-1 | vs | Chemotherapy | **0.34 (0.22 to 0.55)** |
|  |  | Anti-PD-L1 | vs | Anti-PD-L1 plus chemotherapy | 0.25 (0.06 to 1.01) |
|  |  | Anti-PD-1 plus chemotherapy | vs | Anti-PD-L1 plus chemotherapy | 1.46 (0.32 to 6.87) |
|  |  | Anti-PD-1 | vs | Anti-PD-L1 plus chemotherapy | **0.27 (0.11 to 0.69)** |
|  |  | Anti-PD-1 plus chemotherapy | vs | Anti-PD-L1 | 5.79 (1.03 to 31.89) |
|  |  | Anti-PD-1 | vs | Anti-PD-L1 | 1.06 (0.32 to 3.59) |
|  |  | Anti-PD-1 | vs | Anti-PD-1 plus chemotherapy | **0.18 (0.05 to 0.75)** |
|  | irAE 1-5 | Anti-PD-L1 plus chemotherapy | vs | Chemotherapy | 1.95 (0.68 to 5.64) |
|  |  | Anti-PD-L1 | vs | Chemotherapy | 1.53 (0.35 to 6.72) |
|  |  | Anti-PD-1 plus chemotherapy | vs | Chemotherapy | **2.92 (1.15 to 7.10)** |
|  |  | Anti-PD-1 | vs | Chemotherapy | **4.18 (1.51 to 13.95)** |
|  |  | Anti-PD-L1 | vs | Anti-PD-L1 plus chemotherapy | 0.79 (0.13 to 4.85) |
|  |  | Anti-PD-1 plus chemotherapy | vs | Anti-PD-L1 plus chemotherapy | 1.50 (0.36 to 5.84) |
|  |  | Anti-PD-1 | vs | Anti-PD-L1 plus chemotherapy | 2.14 (0.50 to 10.89) |
|  |  | Anti-PD-1 plus chemotherapy | vs | Anti-PD-L1 | 1.91 (0.33 to 10.48) |
|  |  | Anti-PD-1 | vs | Anti-PD-L1 | 2.72 (0.47 to 19.17) |
|  |  | Anti-PD-1 | vs | Anti-PD-1 plus chemotherapy | 1.43 (0.38 to 6.72) |
|  | irAE 3-5 | Anti-PD-L1 plus chemotherapy | vs | Chemotherapy | 1.81 (0.13 to 25.60) |
|  |  | Anti-PD-L1 | vs | Chemotherapy | 2.75 (0.19 to 39.31) |
|  |  | Anti-PD-1 plus chemotherapy | vs | Chemotherapy | 2.79 (0.52 to 15.09) |
|  |  | Anti-PD-1 | vs | Chemotherapy | 3.08 (0.48 to 28.67) |
|  |  | Anti-PD-L1 | vs | Anti-PD-L1 plus chemotherapy | 1.52 (0.04 to 64.36) |
|  |  | Anti-PD-1 plus chemotherapy | vs | Anti-PD-L1 plus chemotherapy | 1.54 (0.07 to 35.94) |
|  |  | Anti-PD-1 | vs | Anti-PD-L1 plus chemotherapy | 1.69 (0.07 to 57.65) |
|  |  | Anti-PD-1 plus chemotherapy | vs | Anti-PD-L1 | 1.02 (0.04 to 23.69) |
|  |  | Anti-PD-1 | vs | Anti-PD-L1 | 1.11 (0.05 to 38.69) |
|  |  | Anti-PD-1 | vs | Anti-PD-1 plus chemotherapy | 1.10 (0.09 to 18.71) |
| Second-line or higher | trAE 1-5 | Chemotherapy | vs | Placebo | **5.31 (2.81 to 10.05)** |
|  |  | Anti-PD-L1 | vs | Placebo | 1.62 (0.80 to 3.24) |
|  |  | Anti-PD-1 | vs | Placebo | **2.04 (1.14 to 3.70)** |
|  |  | Anti-PD-L1 | vs | Chemotherapy | **0.31 (0.17 to 0.56)** |
|  |  | Anti-PD-1 | vs | Chemotherapy | **0.38 (0.27 to 0.54)** |
|  |  | Anti-PD-1 | vs | Anti-PD-L1 | 1.26 (0.66 to 2.44) |
|  | trAE 3-5 | Chemotherapy | vs | Placebo | **14.51 (5.10 to 41.39)** |
|  |  | Anti-PD-L1 | vs | Placebo | 2.95 (0.94 to 9.27) |
|  |  | Anti-PD-1 | vs | Placebo | **3.37 (1.25 to 8.97)** |
|  |  | Anti-PD-L1 | vs | Chemotherapy | **0.20 (0.08 to 0.52)** |
|  |  | Anti-PD-1 | vs | Chemotherapy | **0.23 (0.14 to 0.39)** |
|  |  | Anti-PD-1 | vs | Anti-PD-L1 | 1.14 (0.41 to 3.18) |

Note: significant results are in bold.

irAEs: immune-related adverse events; NSCLC: non-small cell lung cancer; trAEs: treatment-related adverse events.
